# Traditional Chinese medicine in treating ischemic stroke by modulating mitochondria: A comprehensive overview of experimental studies

**DOI:** 10.3389/fphar.2023.1138128

**Published:** 2023-03-22

**Authors:** Lu Liu, Daohong Chen, Ziyang Zhou, Jing Yuan, Ying Chen, Mingsheng Sun, Mengdi Zhou, Yi Liu, Shiqi Sun, Jiao Chen, Ling Zhao

**Affiliations:** ^1^ Acupuncture and Tuina School, Chengdu University of Traditional Chinese Medicine, Chengdu, Sichuan, China; ^2^ Acupuncture and Chronobiology Key Laboratory of Sichuan Province, Chengdu, Sichuan, China

**Keywords:** traditional Chinese medicine, mitochondria, molecular mechanism, review, ischemic stroke

## Abstract

Ischemic stroke has been a prominent focus of scientific investigation owing to its high prevalence, complex pathogenesis, and difficulties in treatment. Mitochondria play an important role in cellular energy homeostasis and are involved in neuronal death following ischemic stroke. Hence, maintaining mitochondrial function is critical for neuronal survival and neurological improvement in ischemic stroke, and mitochondria are key therapeutic targets in cerebral stroke research. With the benefits of high efficacy, low cost, and high safety, traditional Chinese medicine (TCM) has great advantages in preventing and treating ischemic stroke. Accumulating studies have explored the effect of TCM in preventing and treating ischemic stroke from the perspective of regulating mitochondrial structure and function. In this review, we discuss the molecular mechanisms by which mitochondria are involved in ischemic stroke. Furthermore, we summarized the current advances in TCM in preventing and treating ischemic stroke by modulating mitochondria. We aimed to provide a new perspective and enlightenment for TCM in the prevention and treatment of ischemic stroke by modulating mitochondria.

## 1 Introduction

Stroke is a devastating disease with high disability and mortality rates worldwide (Ajoolabady et al., 2021). Approximately 7,95,000 people suffer from either new or recurrent strokes annually, increasing the economic burden on the family and society (Virani et al., 2020). Most strokes are ischemic, accounting for approximately 87% of all strokes. Ischemic stroke causes brain tissue necrosis due to narrowing or occlusion of the blood supply arteries (carotid and vertebral arteries) and insufficient blood supply to the brain (Shao et al., 2020).

In patients with ischemic stroke, a significant decline in focal cerebral blood flow causes glucose and oxygen deprivation (OGD). Mitochondrial dysfunction is an early and initiating event in OGD following ischemia. It is increasingly evident that it plays a critical role in the onset, development, and pathology of ischemic stroke (Song et al., 2022). During ischemic stroke, OGD causes adenosine triphosphate (ATP) consumption and Na^+^/K^+^ ATPase pump failure, resulting in neuronal membrane depolarization and excessive glutamate release. Excessive Ca^2+^ influx can cause reactive oxygen species (ROS) production and mitochondrial dysfunction, such as an imbalance in mitochondrial dynamics, mitochondrial-induced apoptosis, mitochondrial biogenesis dysfunction, and mitophagy over-activation. These cellular processes eventually lead to neuronal cell death.

The most effective treatment for acute ischemic stroke is reperfusion therapy, which aims to restore blood flow and oxygen levels before neuronal damage occurs. Tissue plasminogen activator (tPA) is the only thrombolytic agent approved by the U.S. Food and Drug Administration for patients with acute ischemic stroke (Alkahtani et al., 2023). However, the narrow treatment window and the risk of complications limit its clinical application (Zhang et al., 2022; Li et al., 2022c). Additionally, tPA can cause mitochondria to produce excessive ROS, exacerbating cell damage (Giorgi et al., 2018). New therapeutic agents are required to address the paucity of stroke management approaches. Increasing evidence suggests that maintaining mitochondrial function is critical for neuronal survival and neurological improvement in ischemic stroke, and mitochondria are the key therapeutic targets in cerebral stroke research (Andrabi et al., 2020; Zhou et al., 2021; Zhong et al., 2022). Therefore, a promising treatment option for ischemic stroke that targets mitochondria is needed.

Chinese herbs and acupuncture are essential components of traditional Chinese medicine (TCM). In clinical trials and basic research, Chinese herbs and acupuncture have demonstrated therapeutic effects in preventing and treating ischemic stroke (Zhang et al., 2021; Song et al., 2022). Further studies reported that Chinese herbs and acupuncture could prevent and relieve cerebral ischemia injury *in vivo* and *in vitro* and have neuroprotective effects by modulating the mitochondrial respiratory chain (Zhong et al., 2009), increasing mitochondrial biogenesis (Sun et al., 2021), inhibiting the mitochondrial apoptotic pathway (Bai et al., 2020) and attenuating excessive mitophagy (Ting et al., 2017). However, only a few studies have comprehensively reviewed these studies hindering the elucidation of the mechanism of action of TCM and the development of clinical applications. Moreover, the existing review, published 2 years ago, only summarized the effects of Chinese herbs on mitochondrial permeability transition pore (mPTP) overopening-induced ischemic neuron apoptosis. Nonetheless, the regulatory effects of TCM on mitochondria in the treatment of ischemic stroke are multifaceted and acupuncture, an integral aspect of TCM, appears to be overlooked in the treatment of ischemic stroke by restoring mitochondrial function.

Understanding the molecular mechanisms of the mitochondria involved in ischemic stroke is crucial to identify potential interventional targets. Thus, we first discuss the role of mitochondria in ischemic stroke. We subsequently summarized the recent advances in TCM in preventing and treating ischemic stroke by regulating mitochondria. We aimed to provide a new perspective and insight into the use of TCM in treating ischemic stroke by improving mitochondrial structure and function.

## 2 The role of mitochondria in ischemic stroke

### 2.1 Ischemic stroke cascade involves mitochondrial function and structure changes

#### 2.1.1 Ischemic stroke cascade involves mitochondrial function changes

Mitochondria produce the majority of ATP *via* the mitochondrial respiratory chain and oxidative phosphorylation to meet the high-energy demands of neurons in the brain that are extremely sensitive to ischemia and hypoxia. Within minutes of the onset of cerebral ischemia, ATP depletion deactivates the Na^+^/K^+^ ATPase pump, causing excessive glutamate release into the extracellular fluid (Sarmah et al., 2020). Overactivation of glutamate receptors, such as N-methyl-D-aspartate-receptor, α-amino-3-hydroxy-5-methyl-4-isox-azolepropionic acid receptor, and kainic acid receptor, results in Ca^2+^ influx and accumulation into cells (Hu et al., 2018; Engin and Engin, 2021; Guo and Ma, 2021). A large Ca^2+^ influx leads to a series of events ranging from mPTP opening and dissipation of mitochondrial membrane potential (MMP) to the release of cytochrome c (Cyt-c) or apoptosis-inducing factor (AIF), thus activating effector caspases and eventually causing neuronal death (Anzell et al., 2018; Li et al., 2020). Concomitantly, decreased ATP depletes nicotinamide adenine dinucleotide (NAD^+^), and the reduced NAD^+^ drives mitochondria to the vicinity of the endoplasmic reticulum to form mitochondria-associated endoplasmic reticulum membranes (MAMs). Moreover, certain MAM-related proteins join mPTP to regulate its opening, an important marker of cerebral cell death during ischemia/reperfusion (I/R).

In addition to energy production, mitochondria are the primary producers of intracellular ROS and are sites of eukaryotic oxidative metabolism. Disrupting mitochondrial electron transport increases ROS generated during cerebral ischemia, particularly during reperfusion (He et al., 2020). Further, this excess ROS affects mitochondrial function and promotes neuroinflammation and neuronal apoptosis after oxygen-glucose deprivation/reoxygenation (OGD/R) (Yang et al., 2021).

#### 2.1.2 Ischemic stroke cascade involves mitochondrial structure changes

In addition to the function of mitochondria, their structure also plays an important role in the pathophysiological process of ischemic stroke. Mitochondria are highly dynamic cellular organelles that can change the shape, size, position, and integrity of mitochondrial DNA (mtDNA) through highly coordinated fission, fusion, and transport to tactical locations. The imbalance of mitochondrial fission and fusion after stroke may increase mitochondrial fragmentation, cause aberrant mitochondrial morphology, and disrupt mitochondrial homeostasis, leading to mitochondrial dysfunction and ultimately triggering neuronal death (Li et al., 2022). Additionally, mutation of gene-encoded subunits in mtDNA results in increased ROS generation, which makes mtDNA more susceptible to mutations than nuclear DNA (Zhang et al., 2022). Researchers have reported that the frequency of mtDNA mutations was significantly higher in the brains of patients with ischemic stroke (Luan et al., 2021). In summary, the ischemic stroke cascade involves changes in mitochondrial function and structure, indicating that mitochondrial structure and function play a critical role in the pathogenesis of ischemic stroke.

### 2.2 Mitochondrial biogenesis in ischemic stroke

Mitochondrial biogenesis is a multifaceted process involving the coordinated regulation of mitochondrial and nuclear transcription factors. Peroxisome proliferator-activated receptor γ coactivator-1α (PGC-1α) is a major regulator of mitochondrial biogenesis. During ischemic stroke, PGC-1α is first activated by upstream AMP-activated protein kinase (AMPK) phosphorylation and sirtuin 1 (SIRT1) acetylation (Kaarniranta et al., 2018), which then interacts with downstream nuclear respiratory factor 1/2 (NRF1/2), taking part in the expression of nuclear and mitochondrial respiratory factors. The binding of NRF1 to the promoter of the mitochondrial transcription factor A (TFAM) gene is enhanced under oxidative stress. Activated TFAM promotes mtDNA copying, transcription, and related protein synthesis, ultimately inducing mitochondrial biogenesis (Ryoo and Kwak, 2018). Additionally, two mitochondrial proteins, uncoupling protein 2 and superoxide dismutase 2, both regulated by PGC1-α, play a pivotal role in counteracting the damaging effects elicited by excessive oxidative stress in ischemic stroke (Chen et al., 2011). Peroxisome proliferator-activated receptor gamma agonists can upregulate PGC-1α, NRF1, TFAM, and cytochrome c oxidase subunits I and IV and enhance mitochondrial biogenesis in ischemic stroke (Yang et al., 2018). This indicated that mitochondrial biogenesis exerted a protective effect by enhancing the signal transduction pathways upstream of mitochondrial biogenesis.

Generally, mitochondrial biogenesis plays an important role as an endogenous protective mechanism in ischemic stroke. Therefore, boosting the signal transduction pathways upstream of mitochondrial biogenesis, such as the PGC-1α signaling cascade, may become a novel therapeutic strategy against ischemic brain damage.

### 2.3 Mitochondrial dynamics in ischemic stroke

Mitochondrial dynamics include fission and fusion. Mitochondrial fission allows damaged mitochondria to separate, leading to their subsequent elimination by mitophagy. The production of one or more daughter mitochondria is highly dependent on dynamin-related protein 1 (Drp1). Mitochondrial fusion facilitates the complementation of neighboring mitochondria, enabling the survival of damaged mitochondria (Zhou et al., 2021). It is a two-step process that requires the fusion of outer and inner mitochondrial membranes, mediated by mitofusins-1/mitofusins-2 (Mfn1/2) and optic atrophy 1 (Opa1), respectively.

The interaction between calcium overload, ROS production, and mPTP increases mitochondrial fission and decreases mitochondrial fusion in ischemic stroke (Zhou et al., 2021). Although increased mitochondrial fission during hypoxia may increase mitochondrial energy production, which is beneficial for maintaining neural function after stroke (Quintana et al., 2019), inducing excessive mitochondrial fission is harmful to neurons (Zhang et al., 2020). Excessive mitochondrial fission affects intracellular calcium homeostasis, exacerbates excitotoxicity, and accelerates neuronal death after ischemic stroke (Zhou et al., 2021). Researchers have observed increased levels of Drp1 in mice subjected to cerebral ischemia and reperfusion injury. After the knockdown of Drp1, oxidative stress, mitochondrial ROS production, and infarct volume decrease, contributing to the survival of neurons in cerebral ischemia (He et al., 2020). Mitochondrial fusion can repair damaged mitochondria and produce additional energy by upregulating the activity of ATP synthase through mitochondrial cristae remodeling (Cohen and Tareste, 2018). The levels of mitochondrial fusion proteins, such as Mfn-1/Mfn-2 and Opa1, decrease after cerebral ischemia (Rutkai et al., 2019). However, hypoxia-induced apoptosis improved when Mfn-2 was restored (Zhou et al., 2022).

In summary, inhibiting excessive mitochondrial fission, promoting mitochondrial fusion, and restoring the balance of mitochondrial dynamics are beneficial for ischemic stroke recovery. Maintaining this balance can serve as a target for treating ischemic stroke.

### 2.4 Mitophagy in ischemic stroke

Mitophagy is a type of selective autophagy in which damaged or dysfunctional mitochondria are removed. In ischemic stroke, mitophagy could be predominantly mediated by the PINK1/Parkin pathway, Bcl-2/E1B-19 KD-interacting protein 3 (BNIP3), NIP3-like protein X (NIX, also known as BNIP3L), and FUN14 domain containing 1 (FUNDC1). Shen et al. demonstrated that mitophagy could protect brain cells from ischemic injury during the ischemic phase of stroke (Shen et al., 2021). In contrast, mitophagy serves as a double-edged sword when the brain suffers from reperfusion injury. Activating mitophagy to clear excessively aggregated and damaged mitochondria reduces neuronal damage caused by cerebral I/R injury (Li et al., 2018; Wang and Xu, 2020; Wu et al., 2021). However, some studies have shown that inhibiting excessive mitophagy can protect against cerebral I/R injury in middle cerebral artery occlusion (MCAO) rats (Lan et al., 2018; Jakic et al., 2019). Inhibition of excessive mitophagy could exert neuroprotective effects against neuronal death caused by chronic cerebral hypoperfusion (Su et al., 2018).

Mitophagy is important for the pathogenesis of cerebral I/R damage. Regulation of mitophagy could exert neuroprotective effects in ischemic stroke, although some issues regarding its role in ischemic stroke remain unclear. It would be meaningful to explore the role of mitophagy in treating I/R.

### 2.5 Proteins associated with mitochondria-dependent apoptosis in ischemic stroke

Apoptosis is a planned or controlled cell death triggered by mitochondrial malfunction through intrinsic and extrinsic pathways. Mitochondria are associated with many apoptosis-related proteins, suggesting that they are crucial for cell death following I/R (Yang et al., 2018). Many studies have revealed that B cell lymphoma (BCL-2) family proteins regulate neuronal death in cerebral ischemic stroke (Ader et al., 2019). After I/R, apoptotic members of the Bcl-2 protein family (e.g., Bax and Bak) are inserted into the outer mitochondrial membranes, and MMP is significantly downregulated. Another decisive step in the apoptotic cascade is related to the mPTP. Transient opening of the mPTP in the mitochondrial inner membrane after I/R causes MMP collapse. Several apoptosis-related proteins (e.g., AIF, Cyt-c, endonuclease G [Endo G], the second mitochondrion-derived activator of caspase/direct inhibitor of apoptosis-binding protein with low pI [Smac/Diablo]) originating in the mitochondria are released into the cytoplasmic matrix (Zhou et al., 2021). After migration to the cytoplasmic matrix, Cyt-c interacts with apoptosis-activating factor-1 (Apaf-1), deoxyadenosine triphosphate (dATP), and procaspase-9 to form the apoptosome, which then activates procaspase-9 and follows with caspase-9 to cleave and activates caspase-3 (Wang et al., 2020). Smac binds to and inhibits inhibitor-of-apoptosis proteins (IAPs), which normally inhibit procaspase activation and caspases activity (Zhao et al., 2020). AIF can trigger caspase-independent chromatin condensation and large-scale DNA breakage (Yang et al., 2017) and functions as a mitochondrial effector of apoptotic cell death following translocation from mitochondria to the nucleus (Guida et al., 2019).

Overall, modulating the expression of apoptotic members of the Bcl-2 protein family and preventing translocation of AIF, Cyt-c, and Smac from the mitochondria into the cytoplasmic matrix can serve as targets for the treatment of ischemic stroke.

## 3 Progress in ischemic stroke prevention and treatment using TCM that regulates mitochondria

Based on the above summary, we identified several targets for treating ischemic stroke from the mitochondrial perspective. In clinical and experimental studies, TCM has demonstrated significant efficacy in preventing and treating ischemic stroke. The mechanisms of action of TCM have also been gradually revealed in recent years. Many studies have revealed that TCM exerts therapeutic effects on ischemic stroke by regulating the mitochondria. Therefore, we summarized the literature on acupuncture, herbal extracts, effective TCM compounds, and TCM prescriptions in preventing and treating ischemic stroke and attempted to further clarify the molecular mechanisms of TCM in improving ischemic stroke from the perspective of regulating mitochondria.

### 3.1 Acupuncture and its molecular mechanisms for regulating mitochondria in ischemic stroke

#### 3.1.1 Acupuncture pretreatment for regulating mitochondria in ischemic stroke

The MCAO group exhibited apparent mitochondrial structural abnormalities, including a reduction in mitochondrial volume and number, swelling, vacuolization, formation of autophagosomes and lysosomes, and broken/irregular/disappeared inner membranes and cristae. However, 5–7 consecutive days of electroacupuncture (EA) pretreatment reduced mitochondrial abnormalities, including an increase in mitochondrial volume and number (Sun et al., 2021), less swelling (Tian et al., 2022), a relatively integrated membrane and cristae (Zhang et al., 2018), and a reduction in the number of autolysosomes (Tian et al., 2022). Elevated radical generation (Sun et al., 2021), attenuated MMP levels (Mao et al., 2020; Sun et al., 2021; Tian et al., 2022), and reduced citrate synthase (Sun et al., 2021) were detected in the MCAO group 24 h after reperfusion, compared with those in the control group. These trends could be reversed by EA pretreatment. Additionally, researchers reported that five consecutive days of EA pretreatment at the Baihui (DU20) acupoint induced neuronal protection by inhibiting the expression (Zhang et al., 2017; Zhang et al., 2018) and translocation (Zhang et al., 2018) of mitochondrial Drp1 in rats with focal cerebral IR injury. Meanwhile, EA pretreatment at the DU20 and Shuigou (DU26) acupoints for 5 days was applied to treat cerebral I/R injury in rats and exerted neuroprotective effects by inhibiting the autophagy-related p-ULK1/FUNDC1 pathway (Mao et al., 2020; Tian et al., 2022). EA pretreatment at the DU20 acupoint induced cerebral ischemic tolerance, increased the expression of NRF-1, TFAM, and mtDNA levels, and further promoted mitochondrial biogenesis by activating CB1R-dependent PGC-1α (Sun et al., 2021). Sun et al. found that the release of Cyt-c in the cytoplasm (Cyto-Cyt-c) was reduced in the EA group 24 h after reperfusion compared with that in the I/R mice group induced by MCAO (Sun et al., 2021). Their findings were consistent with another previous study that also found a significant decrease in Cyto-Cyt-c levels in the EA group compared with the IR group at 6, 24, and 48 h after reperfusion (Zhang et al., 2018).

**FIGURE 1:**
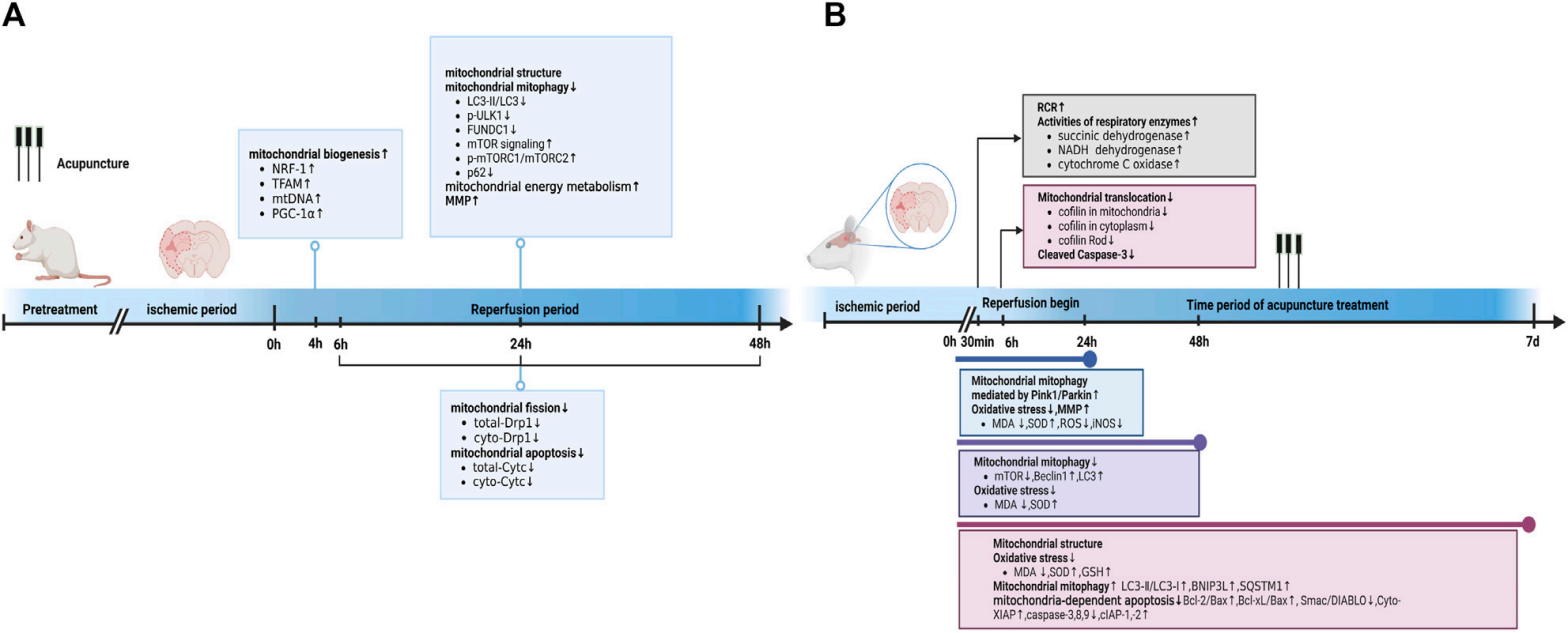
Acupuncture prevented and treated ischemic stroke by regulating mitochondria. **(A)** Acupuncture prevented ischemic stroke by regulating mitochondria at different time point in reperfusion stage. **(B)** Acupuncture treated ischemic stroke by regulating mitochondria at different time point of acupuncture treatment. Abbreviations: RCR, respiratory control ratio; LC3-I/II, Light chain 3I/II; GSH, glutathione; SQSTM1, Sequestosome-1; iNOS, inducible nitric oxide synthase.

In summary, EA pretreatment promoted mitochondrial biogenesis 4 h after reperfusion. At 6, 24, and 48 h after reperfusion, EA pretreatment inhibited mitochondrial fission and apoptosis by decreasing mitochondrial Drp1 and Cyto-Cyt-c levels, respectively. Moreover, after 24 h of reperfusion, EA pretreatment reversed mitochondrial structural abnormalities, inhibited the autophagy-related p-ULK1/FUNDC1 pathway, attenuated radical generation, elevated MMP levels, and increased mitochondrial energy metabolism. Specific mechanisms are shown in Table 1; Figure 1A.

**TABLE 1:** The molecular mechanism of acupuncture in the treatment of ischemic stroke by targeting mitochondria.

Acupuncture method	Animals	Gender	Weight	Animal model	Prevention/treatment	Time period	Insertion depth, Stimulator parameters	Acupoints	Mechanisms	References
EA	SD rat	Male	200–250 g	MCAO (2 h)/R (24 h)	Pretreatment	Pretreatment for 5 days, q.d. 30 min per day	Baihui (DU20):2 mm,Shuigou (DU26):1 mm; alternating frequency of 2/50 Hz; A slight rat limb tremor reflects an appropriate stimulus intensity.	Baihui (DU20) and Shuigou (DU26)	MMP↑,LC3-II/LC3-I↓,p-ULK1↓,FUNDC1↓, mTOR signaling↑	Tian et al. (2022)
EA	SD rat	Male	220–250 g	MCAO (2 h)/R (24 h)	Pretreatment	Pretreatment for 5 days, q.d. 30min per day	Baihui (DU20):1 mm; Shuigou (DU26):1 mm; density-sparse wave; intensity of 1 mA	Baihui (DU20) and Shuigou (DU26)	MMP↑, FUNDC1↓, LC3-II/I↓, p-mTORC1/mTORC2↑,p62↓	Mao et al. (2020)
EA	SD rat	Male	300 ± 20 g	MCAO (2 h)/R (6,24,48 h)	Pretreatment	Pretreatment for 5 days, q.d. 30 min per day	Baihui (DU20): 2mm; frequency, 2/15 Hz; intensity of 1 mA	Baihui (DU20)	TUNEL-positive neurons↓, total Drp1↓, Mito-Drp1↓, total-cyt-c↓, cyto-cyt-c ↓	Zhang et al. (2018a)
EA	C57BL6j mice	Male	25–30 g	MCAO (1 h)/R (4 h,24 h)	Pretreatment	30 min	frequency of 2/15 Hz, intensity of 1 mA	Baihui (DU20)	cyto-cyt c↓,COXⅣ↑,	Sun et al. (2021)
									ROS↓,MMP↑, citrate synthase↑,NRF-1↑,	
									TFAM↑,mtDNA↑,PGC-1α↑,TUNEL-positive neurons↓	
EA	Wistar rat	Male	250–300 g	MCAO (2 h)/R (6,24,48 h)	Pretreatment	Pretreatment for 5 days, q.d. 30 min per day	frequency of 2/15 Hz,	Baihui (DU20)	Drp1↓,TUNEL-positive neurons↓	Zhang et al. (2017b)
							intensity of 1 mA			
EA	SD rat	Male	300–350 g	MCAO (30 min)/R (7 days)	treatment	7 days, q.d. 25 min per day	Baihui (GV20) 4 mm; Fengfu (GV16):7.5 mm; 150-μs pulse width; intensity of 2.7–3.0 mA	Baihui (GV20) and Fengfu (GV16)	cytosolic p-p38 MAPK/p38 MAPK ↑, Cytosolic GFAP↓, cytosolic p-CREB/CREB↑, Cytosolic Bcl-2↑, Cytosolic Bax↓, Cytosolic Bcl-xL↑, cytosolic Bcl-2/Bax↑, Bcl-xL/Bax↑Mitochondrial Bcl-2↑,Mitochondrial Bax ↓, mitochondrial Bcl-xL ↑,mitochondrial Bcl-2/Bax↑, mitochondrial Bcl-xL/Bax ↑,Mitochondrial and cytosolic Smac/DIABLO↓,Cytosolic XIAP↑, Cytosolic cleaved caspase-3↓	Cheng et al. (2015)
EA	SD rat	Male	150–180 g	MCAO (90 min)/R (3 days)	treatment	30 min/time,2 times per day, lasting for 3 days	Baihui (DU20):3 mm; Qihai (RN6):3 mm; 2 Hz; intensity of 1 mA	Baihui (DU20) and Qihai (RN6)	Bcl-2↑,Bcl-xL↑,cIAP-1↑, cIAP-2↑,caspase-3↓,	Kim et al. (2013)
									caspase-9↓,caspase-8↓,	
									TUNEL-positive cells ↓, Cleaved PLCγ1↓, Dr5↓	
EA	SD rat	Not mentioned	200–250 g	4-VO(3 h)/R (48 h)	treatment	5 times within 48 h, 20min/time	Baihui (DU20):2 mm; Mingmen (DU4):5–7 mm	Baihui (DU20),Mingmen (DU4),Zusanli (ST36)	mTOR↓,Beclin1↑,LC3↑,IL-6↓, TNF-α↓,IL-1β↓, MDA ↓,SOD↑	Ting et al. (2017)
							Zusanli (ST36):7mm; frequency of 40–50 Hz; A slight rat limb tremor reflects an appropriate stimulus intensity.			
EA	SD rat	Male	280–300 g	MCAO/R (24 h)	treatment	EA at 5 min and 6 h after reperfusion, 30min/time	frequency of 4/20 Hz; intensity of 1 mA.	Baihui (DU20) and Shenting (DU24)	Cleaved Caspase-3↓,	Chen et al. (2021)
									TUNEL-positive cells↓,	
									Cofilin Rod↓, MAP2 degradation↓, cofilin in mitochondria and cytoplasm↓	
EA	SD rat	Male	200–220 g	MCAO (90min)/R (24 h)	treatment	30 min EA treatment	Renzhong (DU26):1 mm	Baihui (DU20) and Renzhong (DU26)	RCR↑, succinic dehydrogenase↑, NADH dehydrogenase↑, cytochrome C oxidase↑	Zhong et al. (2009)
							Baihui (DU20):4 mm; disperse-dense waves of 5/20 Hz (28.5 ms/15 ms pulse duration) of frequency; current density of 2–4 mA			
EA	SD rat	Male	220–250 g	MCAO (2 h)/R (24 h)	treatment	2 times within 24 h	Not mentioned	Not mentioned	MMP↑,ATP↑,Opa1↑,Mfn1↑, COX IV↓,VDAC↓,	Wang et al. (2019a)
									TOMM20↓,NOX↓,ROS↓,MDA↓,SOD↑,iNOS↓,3-NT↓,Drp1↑,Parkin↑, Mfn2↑,translocation of Parkin and LC3 from the cytoplasm to mitochondria↑	
EA	SD rat	Male	200–230 g	MCAO (15 min)/R (24 h)	treatment	2 times within 24 h,	frequency of 30–50 Hz; different electric current	Baihui (GV20), Mingmen <	LDH↑, SDH↑,Na + -K+	Tian et al. (2015)
						30min/time	intensities: 5 mA, 3 mA and 1 mA.	(GV4) and Zusanli (ST36).	ATPase↑	
EA	SD rat	Male	280 ± 20 g	MCAO/R (7 days)	treatment	7 days, q.d. 30min per day	Continuous wave of 2/100 Hz and 2–4 V	Baihui (GV20), Shuigou	MDA↓,iron↓,SOD↑,GSH↑,GPX4↑,FTH1↑,Tf↓,TfR↓	Li et al. (2021a)
								(GV26), Sanyinjiao (SP6), and Neiguan (PC6).		
EA	SD rat	Male	300 ± 20 g	MCAO (90 min)/R (7 days)	treatment	7days, q.d. 20min per day	Baihui (DU20)and Shenting (DU24):0.2 cm; disperse-dense waves of 4/20 Hz; current density of 0.5 mA	Baihui (DU20),Shenting (DU24)	LC3-Ⅱ/LC3 Ⅰ↑,BNIP3L↑,	Zhong et al. (2022b)
									SQSTM1↑, TUNEL positive cells↓	

Notes: ↑, upregulate; ↓, downregulate; SD, Sprague-Dawley; MCAO/R, middle cerebral artery occlusion/reperfusion; q. d., once a day; ULK1, Unc-51-like kinase 1; LC3-I/Ⅱ, Light chain 3I/Ⅱ; LC3B-II, light chain 3B II; p62, Sequestosome-1; p38 MAPK, p38 mitogen-activated protein kinases; CREB, cAMP, response element binding protein; PLC γ, 1, phospholipase C γ 1; Dr5, death receptor 5; MAP2, microtubule-associated protein-2; NADH, nicotinamide adenine dineucleotide; RCR, respiratory control ratio; iNOS, inducible nitric oxide synthase; LDH, lactate dehydrogenase; SDH, succinate dehydrogenase; GSH, glutathione; SQSTM1, Sequestosome-1; VDAC, voltage-dependent anion channel; Tomm20, translocase of outer mitochondrial membrane 20 homolog; NOX, oxidase; 3-NT, 3-nitrotyrosine; GPX4, glutathione peroxidase 4; Tf, transferrin; TfR1, transferrin receptor 1, FTH1, ferritin heavy chain 1; GFAP, glial fibrillary acidic protein; mito-Drp1, mitochondrial dynamin-related protein 1; COX IV, cytochrome c oxidase IV; IL-6, interleukin 6; IL-1β, interleukin 1β; TNF-α, Tumor necrosis factor-alpha; p-mTORC1, phosphorylated mTORC1; 4-VO/R, 4-vessel occlusion/reperfusion.

#### 3.1.2 The effect of acupuncture after ischemic stroke in regulating mitochondria

In the MCAO group, the neuronal mitochondria became swollen, the mitochondrial cristae and outer membrane were broken, and EA alleviated the mitochondrial structure abnormalities within 24 h after reperfusion (Li et al., 2021). Acupuncture can alleviate cerebral I/R injury by increasing MMP levels and inhibiting nitro/oxidative stress by downregulating oxidase, ROS, and malondialdehyde (MDA) levels and upregulating superoxide dismutase (SOD) (Ting et al., 2017; Wang et al., 2019). In addition, EA at DU20 and DU26 for 30 min decreased the neurological deficit score, improved the respiratory control ratio, and promoted the activities of respiratory enzymes, including succinic dehydrogenase, NADH dehydrogenase, and cytochrome C oxidase, in MCAO rats (Zhong et al., 2009). These findings are consistent with those reported by Tian et al. (Tian et al., 2015). Tian et al. further pointed out that 3 mA EA could more effectively elevate the activities of succinic dehydrogenase and lactate dehydrogenase compared to 1 mA EA and 5 mA EA in the brain tissue of rats with I/R injury (Tian et al., 2015). Zuo et al. noticed that EA at ZuSanLi (ST36), DU20, and Mingmen (DU4) five times within 48 h after reperfusion could improve cerebral I/R by inhibiting excessive autophagy in neurons (Ting et al., 2017). However, Zhong et al. conducted 7 days of EA treatment at DU20 and Shenting (DU24) after reperfusion and reported that EA could alleviate cerebral I/R injury and improve neural function by promoting BNIP3L mediated autophagic clearance (Zhong et al., 2022). Another study also demonstrated that EA within 24 h after reperfusion decreased the accumulation of damaged mitochondria by increasing Pink1/Parkin-mediated mitophagy clearance to protect cells against neuronal injury in cerebral I/R (Wang et al., 2019). Furthermore, within 24 h after reperfusion, EA at DU20 and Fengfu (DU16) increased the expression of anti-apoptotic Bcl-2, Bcl-Xl, and cellular inhibitor of apoptosis- 1,-2 (cIAP-1, -2), and decreased the activities of caspase-3, -8, and -9 compared with the untreated rats with MCAO (Kim et al., 2013). Similarly, EA at DU20 and DU16 for 7 consecutive days activated p38 MAPK-mediated anti-apoptotic signaling pathways, which ultimately contributed to the prevention of Smac/DIABLO translocation and subsequent restoration of the X-linked inhibitor of apoptosis protein (XIAP) suppression of caspase-3 in the cortical peri-infarct area (Cheng et al., 2015). Another study also found that EA treatment within 6 h of ischemic stroke could attenuate ischemic brain injury and cellular apoptosis by inhibiting mitochondrial translocation of cofilin and caspase-3 cleavage (Chen et al., 2021) (Figure 1B).

In summary, acupuncture treatment and pretreatment could both restore mitochondrial morphology, improve MMP levels, further upregulate mitochondrial energy metabolism, attenuate mitochondrial autophagy, and inhibit mitochondrial-dependent apoptosis. Acupuncture pretreatment promoted mitochondrial biogenesis and inhibited mitochondrial fission. Additionally, acupuncture treatment inhibited oxidative stress, cofilin translocation, and activated mitochondrial autophagy. The detailed mechanisms are shown in Table 1 and Figures 1A,B.

### 3.2 Herbal extract and its molecular mechanisms by regulating mitochondria in treating ischemic stroke

#### 3.2.1 Herbal extract pretreatment in regulating mitochondria of ischemic stroke

Although the clinical treatment of ischemic stroke with a single herb is rare, in recent years, researchers have reported that the individual application of certain herbs has the potential to treat diseases. Mitochondrial ultrastructure injury was partially improved in cerebral I/R rats after pretreatment with *in vitro* cultured *Bos taurus domesticus* Gmelin or *Chrysanthemum morifolium* Ramat. extracts (Lin et al., 2010; Lu et al., 2020). Pretreatment with herbal extracts (e.g., *Astragalus membranaceus* (Fisch.) Bge. combined with *Panax notoginseng* (Burk.) F.H.Chen, *Astragalus membranaceus* (Fisch.) Bge., and *Gardenia jasminoides* (Ellis) alleviated nerve injury after cerebral I/R by improving mitochondrial respiration function and energy metabolism (Huang et al., 2012; Huang et al., 2017; Wang et al., 2021). Previous studies have noted that herbal extracts (including *Pinellia ternata* (Thunb.) Breit., *Rosa laevigata* Michx., *Curcuma Longa* L., *C. morifolium* Ramat., and *Lavandula angustifolia* Mill.) could play a neuroprotective role in the pretreatment of animal models of MCAO by increasing MMP levels and inhibiting mitochondrial oxidative stress (by upregulating SOD, glutathione, glutathione peroxidase catalase, and downregulating MDA, NO, ROS, and peroxynitrite) (Dohare et al., 2008b; Lin et al., 2010; Wang et al., 2012; Zhang et al., 2013; Ye et al., 2016). Recent *in vitro* studies have also shown that *Scrophularia ningpoensis* Hemsl., *Aglaia odorata* Lour., *Spatholobus suberectus* Dunn, and *Arctium lappa* L. roots exert neuroprotective effects by increasing MMP levels and inhibiting mitochondrial oxidative stress in preconditioned OGD/R cell models (Meng et al., 2018; Park et al., 2018; Wang K. et al., 2020; Yang et al., 2021). *Lycium barbarum* L. polysaccharide pretreatment decreased cerebral I/R injury in MCAO rats by maintaining mitochondrial fission and fusion balance (upregulating Opa1 and downregulating Drp1) (Liu et al., 2017). Similarly, *Arctium lappa* L. roots ameliorated OGD/R-induced injury by suppressing AMPK/mammalian target of rapamycin (mTOR)-mediated autophagy (Yang et al., 2021). Herbal extracts (such as *P. ternata* (Thunb.) Breit., *R. laevigata* Michx, *Curcuma Longa* L., *S. ningpoensis* Hemsl., *L. barbarum* L. polysaccharides, *Astragalus membranaceus* (Fisch.) Bge., *in vitro* cultured *B. taurus domesticus* Gmelin and *Angelica sinensis* (Oliv.) (Diels) prevented cerebral I/R injury in MCAO animal models by inhibiting the mitochondria-dependent apoptosis pathway. These herbal extracts upregulated the expression of Bcl-2, mitochondrial Cyt-C (Mito-Cyt-c), cytosolic phospho-Bad (p-Bad)/Bad ratios, and mitochondrial p-Bad/Bad. Additionally, they downregulated the expression of Bax, p53, Apaf1, Bax, Bid, Cyt-c, cleaved PARP-1, and active caspase-3, -9, and -8 (Dohare et al., 2008b; Huang et al., 2012; Zhang et al., 2013; Wang et al., 2014; Ye et al., 2016; Cheng et al., 2017; Meng et al., 2018; Lu et al., 2020). Evidence from *in vitro* experiments has demonstrated that *A. odorata* Lour. and *Arctium lappa* L. roots showed a significant protective effect in OGD/R cell models by inhibiting the mitochondria-dependent apoptotic pathway (Wang et al., 2020; Yang et al., 2021).

**FIGURE 2:**
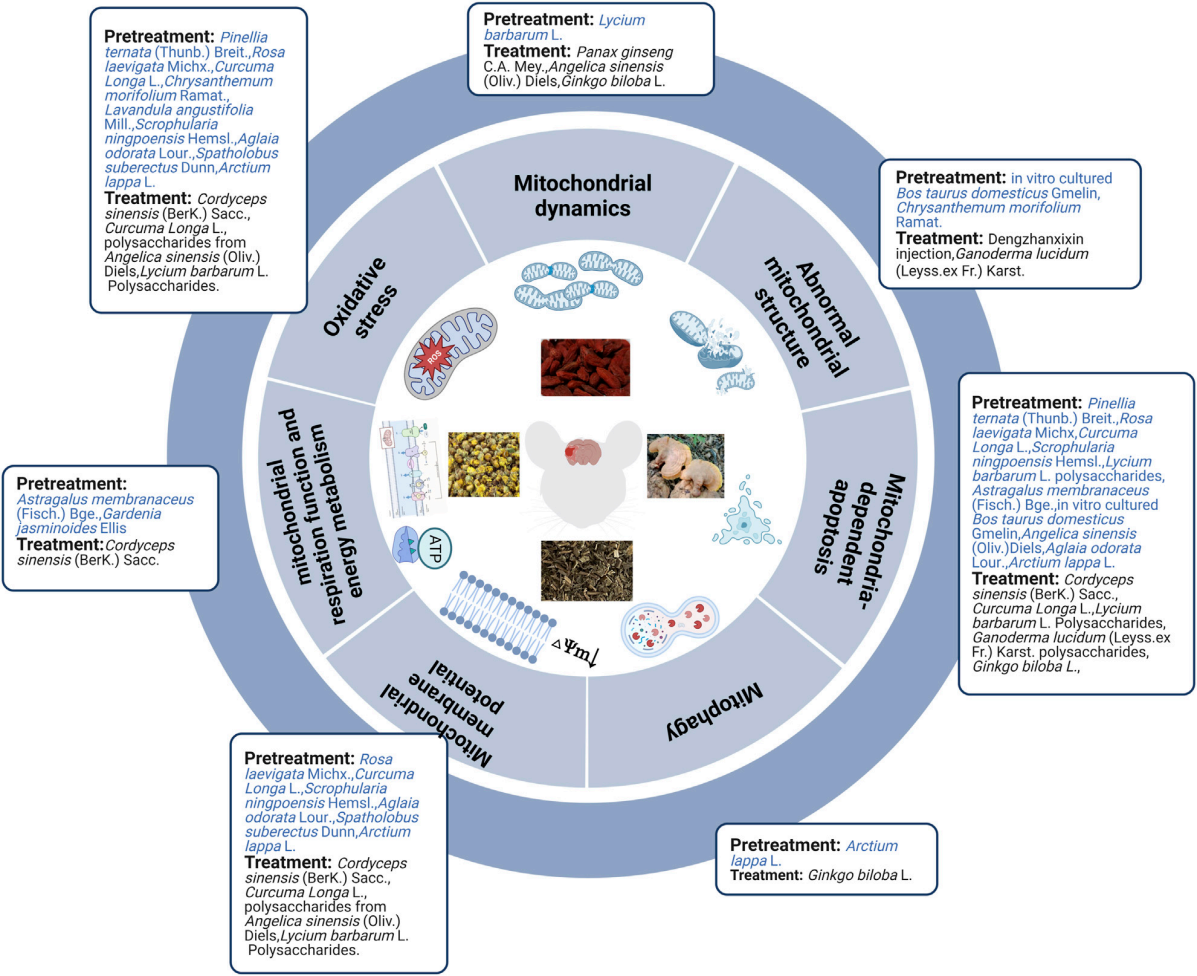
Herbal extract prevented and treated ischemic stroke by regulating mitochondria.

In brief, evidence from *in vivo* and *in vitro* studies indicated that herbal extract pretreatment could ameliorate cerebral ischemia by improving mitochondrial ultrastructure, increasing MMP levels, mitochondrial respiration function, and energy metabolism, maintaining mitochondrial dynamic balance, inhibiting mitochondria-related oxidative stress, autophagy, and mitochondria-dependent apoptosis (Figure 2).

#### 3.2.2 The effect of herbal extract after ischemic stroke in regulating mitochondria

Not only herbal extract pretreatment can alleviate mitochondrial structural abnormalities, but also herbal extract treatment can mitigate these abnormalities. Seven days of Dengzhanxixin injection treatment can improve decreased and unclear mitochondrial cristae observed in the MCAO rat model (An et al., 2021) while *Ganoderma lucidum* (Leyss.ex Fr.) Karst. polysaccharides can alleviate swollen and vacuolized mitochondria observed in OGD/R primary cortical neuronal cells (Zhou et al., 2010). *Cordyceps sinensis* (BerK.) Sacc. extract improved ATP levels and mitochondrial complexes I-IV in MCAO rats. *Cordyceps sinensis* (BerK.) Sacc., *Curcuma Longa* L., and polysaccharides from *A. sinensis* (Oliv.) Diels decreased oxygen free radicals, NO, ROS, peroxynitrite, glutathione peroxidase, SOD, and Ca2^+^ and increased MMP and MDA levels in MCAO rats (Dohare et al., 2008a; Lei et al., 2014; Bai et al., 2020). These findings are consistent with *in vitro* studies of *C. sinensis* (BerK.) Sacc. extract, *L. barbarum* L. polysaccharides, and polysaccharides from *A. sinensis* (Oliv.) Diels in alleviating OGD/R injury (Lei et al., 2014; Shi et al., 2017; Zhao et al., 2017; Bai et al., 2020). Additionally, combining *Panax ginseng* C.A. Mey. and *A. sinensis* (Oliv.) Diels partially attenuated cerebral injury by ameliorating Drp1-mediated mitochondrial fission (downregulating Drp1) *in vivo* and *in vitro* (Hu et al., 2020). However, *Ginkgo biloba* L. extract upregulated Drp1 and Opa1 *in vivo* (Li et al., 2019). Researchers found that *G. biloba* L. extract induced autophagy by activating the AMPK/mTOR pathway (Li et al., 2019). Both *in vivo* and *in vitro* experiments, including *C. sinensis* (BerK.) Sacc., *Curcuma Longa* L., *L. barbarum* L. polysaccharides, *G. lucidum* (Leyss.ex Fr.) Karst. polysaccharides, and extract of *G. biloba L.*, exhibited obvious neuroprotective effects in MCAO rats, and the OGD/R cell model by inhibiting mitochondrial-dependent apoptosis (Dohare et al., 2008a; Zhou et al., 2010; Shi et al., 2017; Zhao et al., 2017; Li et al., 2019; Bai et al., 2020) (Figure 2).

**FIGURE 3:**
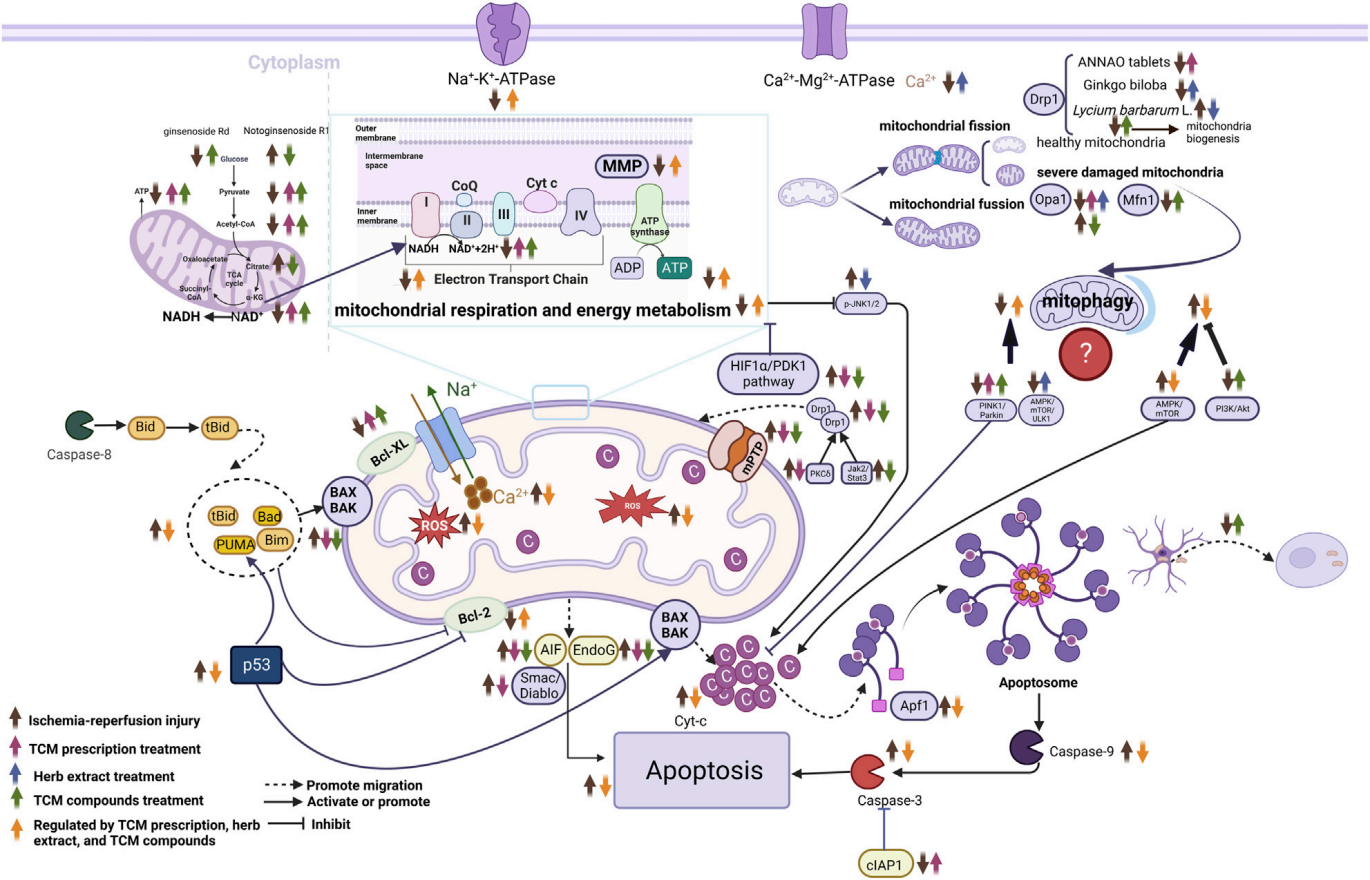
Molecular mechanisms of herbal extract, TCM compounds and TCM prescriptions in treating ischemic stroke from the prospective of mitochondria. ↑, upregulate; ↓, downregulate; Abbreviations: NAD^+^, nicotinamide adenine dinucleotide; Endo G, endonuclease G; Apaf-1, apoptosis activating factor-1; AIF, apoptosis-related proteins; Mfn1, mitofusins-1; Opa1, optic atrophy 1; Smac/Diablo, the second mitochondrion-derived activator of caspase/direct inhibitor of apoptosis-binding protein with low pI; cIAP-1, cellular inhibitor of apoptosis- 1; Cyt-c, cytochrome C; mPTP, mitochondrial permeability transition pore; MMP, mitochondrial membrane potential; AMPK, AMP-activated protein kinase; mTOR, mammalian target of rapamycin; Drp1, dynamin-related protein 1; HIF1α/PDK1, Hypoxia-Inducible Factor 1-Alpha/Pyruvate Dehydrogenase Kinase 1; NADH, Nicotinamide adenine dineucleotide; JNK, c-Jun N-terminal kinase; ROS, reactive oxygen species; TCA, tricarboxylic acid.

Overall, herbal extract pretreatment and treatment could alleviate abnormal mitochondrial structure; improve MMP, mitochondrial energy metabolism, mitochondrial respiration function, and mitochondrial fusion; and inhibit oxidative stress, mitochondrial fission, and mitochondrial-dependent apoptosis. Furthermore, herbal extract pretreatment suppressed AMPK/mTOR-mediated mitophagy, whereas herbal extract treatment induced autophagy by activating the AMPK/mTOR pathway and promoting mitochondrial fission. Specific mechanisms are shown in Table 2; Figure 3.

**TABLE 2:** The molecular mechanism of herbal extracts in the treatment of ischemic stroke by targeting mitochondria.

Herbal extracts	Cell lines and cell models	Animals	Gender	Weight	Animal model	Routes	Dose	Prevention/treatment	Time periods	Mechanisms	References
*In vitro* cultured *Bos taurus domesticus* Gmelin extract	-	SD rat	Male	240–280 g	MCAO (90 min)/R (24 h)	intragastric administration	25,50,100 mg/kg	Prevention	Pretreatment for 3 days, q.d.1 h before MCAO and 6 h after MCAO	Bax↓,caspase-9↓,caspase-3↓, Cyto-Cyt-c↓,	Lu et al. (2020)
										Bcl-2↑,Mito-Cyt-c↑	
*Chrysanthemum morifolium* Ramat. extract	-	SD rat	Male	250–300 g	MCAO (90 min)/R (22 h)	intraperitoneal injection	50,100,200 mg/kg	Prevention	90 min before MCAO	SOD↑, MDA↓, ROS↓	Lin et al. (2010)
Extract of *Gardenia jasminoides* Ellis, stir-baked until brown, and fried until carbonized	-	SD rat	Male	250–270 g	MCAO/R (12 h)	intragastric administration	*Gardenia jasminoides* Ellis (0.5.1 g kg^−1^), *Gardenia jasminoides* Ellis stir-baked until brown (0.5.1 g kg^−1^), *Gardenia jasminoides* Ellis fried until carbonized (0.5.1 g kg^−1^)	Prevention	15 min before MCAO	Na^+^-K^+^-ATPase↑,Ca^2+^-Mg^2+^-ATPase↑,ROS↓	Wang et al. (2021b)
*Astragalus membranaceus* (Fisch.) Bge. extract	-	C57BL/6N mice	Male	18–22 g	CCA(20 min)/R (1/24/48 h)	intragastric administration	110 mg/kg	Prevention	at 08:00 (10 mL/kg), 4 days, q.d. before CCA, After suturing the skin, the mice continued to be medicated until awakening from anesthesia.	ATP↑, ADP↑,EC↑,	Huang et al. (2012)
										Na^+^-K^+^ATPase↑, p-JNK1/2↓, Cyt-c↓, caspase-9↓, caspase-3↓	
Extract of *Astragalus membranaceus* (Fisch.) Bge. and *Panax notoginseng* (Burk.) F.H.Chen	-	C57BL/6N mice	Male	18–22 g	CCA(20 min)/R (1/24 h)	intragastric administration	astragalus extract: 110 mg/kg; total panax	Prevention	at 08:00 (10 mL/kg), 4 days, q.d. before CCA, After suturing the skin, the mice continued to be medicated until awakening from anesthesia.	ATP↑,ADP↑, Na^+^-K^+^ATPase↑,p-JNK1/2↓, Cyt-c↓, Caspase-9↓, Caspase-3↓	Huang et al. (2017)
							notoginseng saponins:115 mg/kg				
*Lycium barbarum* L. polysaccharides	-	SD rat	-	200–220 g	CCA(30 min)/R (24/72 h)	intraperitoneal injection	25 mg/kg	Prevention	3 weeks after the induction of diabetes and continued for 4 weeks before MCAO	Opa1↑, Drp1↓	Liu et al. (2017)
Extract from *Pinellia ternata* (Thunb.) Breit.	-	SD rat	Male	250–300 g	MCAO (2 h)/R (24 h)	take orally	5,10,20 mg/kg	Prevention	Pretreatment for 7 days, q.d.	Bcl-2↑, Bax↓, SOD↑, MDA↓	Ye et al. (2016)
*Rosa laevigata* Michx. extract	-	SD rat	Male	250–300 g	MCAO (2 h)/R (24 h)	intragastric administration	50, 100, 200 mg/kg	Prevention	Pretreatment for 7 days, q.d.	SOD↑,GSH↑, T-NOS↓, NO↓, iNOS↓,p53↓, Apaf1↓,Bcl-2↑, Fas↓, FasL↓,Bax↓,Bid↓, Caspase-8↓, Caspase-9↓, Caspase-3↓, Cyt-c↓, MMP-9↓, COX-2↓	Zhang et al. (2013)
*Lavandula angustifolia* Mill. extract	-	Kunming mice	Male	30–34 g	MCAO (2 h)/R (22 h)	intragastric administration	200,100,50 mg/kg	Prevention	Pretreatment for 3 days, q.d.,2 h after MCAO	MDA↓, SOD↑, CAT↑, GSH-Px↑, GSH/GSSG↑, ROS↓	Wang et al. (2012)
Extract of *Arctium lappa* L. roots	SH-SY5Y cells; OGD(4 h)/R (24 h)	-	-	-	-	treated with *Arctium lappa* L.	-	Prevention	12 h before OGD/R	ROS↓, MMP↑, Bax↓,Cyt-c↓,caspase-3↓, Bcl-2↑,Beclin-1↓, LC3-II↓,SQSTM1/p62↑	Yang et al. (2021)
Extract of *Scrophularia ningpoensis* Hemsl.	PC12 cells; OGD(2 h)/R (24 h)	-	-	-	-	treated with *Scrophularia ningpoensis* Hemsl.	12.5 μg/mL	Prevention	Pretreatment for 8/16 h	SOD↑,GSH-Px↑, CAT↑,LDH↓, MMP↑	Meng et al. (2018)
*Aglaia odorata* Lour. extract	PC12 cells; OGD(4 h)/R (24 h)	-	-	-	-	treated with *Aglaia odorata* Lour. extract	-	Prevention	-	ROS↓, cleaved caspase-9/3↓,p53↓, p53/Puma↓, Bcl-2↑	Wang et al. (2020b)
*Spatholobus suberectus* Dunn extract	SH-SY5Y cells; OGD/R	-	-	-	-	treated with *Spatholobus suberectus* Dunn extract	25 or 50 μg/ml	Prevention	Pretreatment for 6 h	MMP↑, caspase-3/7↓	Park et al. (2018)
*Curcuma Longa* L. extract	-	SD rat	Male	200–225 g	MCAO (1 h)/R (24 h)	intraperitoneal injection	250 mg/kg	Prevention	30 min before MCAO	ROS↓, caspase-3↓, cleaved caspase-3↓, Cyt-c↓, p53↓, Bax↓, Bcl-2↑	Dohare et al. (2008b)
*Lycium barbarum* L. polysaccharide	-	ICR mice	Male	20–25 g	MCAO (2 h)/R (24 h)	intragastric administration	10, 20, 40 mg/kg	Prevention	Pretreatment for 7 days, q.d.	caspase-3↓, Bax↓, Cyt-c↓, Bcl-2↑, Caspase-9↓, cleaved PARP-1↓	Wang et al. (2014b)
*Angelica sinensis* (Oliv.)Diels extract	-	SD rat	Male	300–350 g	MCAO (1 h)/R (1/3 days)	intraperitoneal injection	0.25, 0.5, 1 g/kg	Prevention	30 min before MCAO	p-Bad/Bad↑, Cyt-c↓, cleaved caspase-3↓	Cheng et al. (2017)
extract of *Scrophularia ningpoensis* Hemsl.	-	Kunming mice	Male	18–22 g	MCAO (2 h)/R (24 h)	intragastric administration	2.4 g/kg^-1^	Prevention	7 days, q.d., before MCAO	LDH↓, MDA↓,NO↓, Bax↓, Bcl-2↑	Meng et al. (2018)
Dengzhanxixin injection (Dengzhanxixin Zhusheye in Chinese pharmacopoeia)	-	SD rat	Male	270 ± 10 g	MCAO (1.5 h)/R (24 h)	intravenous injection	8.8 mg/kg	treatment	7 days, bid, after MCAO	Infarct volume↓, the survival of neuronal cells↑, modulated the mitochondrial respiratory chain process	An et al. (2021)
*Ganoderma lucidum* (Leyss.ex Fr.) Karst. polysaccharides	primary cortical neuronal cell; OGD(2 h)/R (24 h)	-	-	-	-	treated with *Ganoderma lucidum* (Leyss.ex Fr.) Karst.	0.1,1,10 ug/ml	treatment	30 min before OGD, during the OGD period and afterward until different times after OGD exposure	caspase-3↓,caspase-8↓,caspase-9↓,Bax↓, Bcl-2↑,LDH↓	Zhou et al. (2010)
						polysaccharides					
*Ganoderma lucidum* (Leyss.ex Fr.) polysaccharides	-	SD rat	Male	280–300 g	MCAO/R (1.5 h)	intragastric administration	100,200,400 mg/kg	treatment	Pretreatment for 7 days, q.d., and administration was continued until sacrifice at conclusion of the experiment	TUNEL-positive staining↓	Zhou et al. (2010)
*Cordyceps sinensis* (BerK.) Sacc. extract	-	SD rat	Male	250 ± 10 g,25 ± 5 g	MCAO/R	take orally	1.0 g/kg	treatment	after ischemia for 24 h, every 24 h for three times	OFR↓,Cyt-c↓,ATP↑, COX↑, complexes I-IV↑,Bax↓,caspase-3↓	Bai et al. (2020)
*Curcuma Longa* L. extract	-	SD rat	Male	-	MCAO/R	take orally	500 mg/kg	treatment	After ischemia for 4 h	NO↓, ROS↓, iNOS↓, eNOS↓,Cyt-c↓,Bax↓, Bcl-2↑,caspase-3↓, peroxynitrite↓	Dohare et al. (2008a)
*Angelica sinensis* (Oliv.) Diels polysaccharides	-	SD rat	Male	200–250 g	MCAO (2 h)/R	intravenous injection	200 mg/kg	treatment	2, 26, 50, 74, 98, 122, 146 h after MCAO	SOD↑,GSH-px↑, MDA↓, MMP↑	Lei et al. (2014)
*Cordyceps sinensis* (BerK.) Sacc. extract	Primary BMECs; OGD/R	-	-	-	-	treated with *Cordyceps sinensis* (BerK.) Sacc. extract	5,10 or 20 μg/ml	treatment	12 h before and during OGD	MMP↑,Bax↓,Cyt-c↓, caspase-3↓,Bcl-2↑, caspase-8↓,caspase-9↓	Bai et al. (2020)
*Angelica sinensis* (Oliv.) Diels polysaccharides	PC12 cells; H_2_O_2_-induced	-	-	-	-	treated with *Angelica sinensis* (Oliv.) Diels polysaccharides	0.1–0.8 mg/mL	treatment	Pretreatment for 15 min, 24 h after H_2_O_2_	ROS↓,MMP↑,SOD↑, GSH-Px↑,MDA↓	Lei et al. (2014)
*Lycium barbarum* L. polysaccharide	Primary Cortical Neuron cells; OGD(4 h)/R (24 h)	-	-	-	-	treated with *Lycium barbarum* L. polysaccharide	100 mg/ml	treatment	24 h after OGD	Bad↓, Cyt-c↓, cleaved caspase-3↓, Ca^2+^↓	Shi et al. (2017)
*Lycium barbarum* L. polysaccharide	Primary hippocampal neuronal cells; OGD(4 h)/R (24 h)	-	-	-	-	treated with *Lycium barbarum* L. polysaccharide	10,20,40 mg/l	treatment	at the start of the reperfusion phase	ROS↓, Ca^2+^↓, MMP↑, LDH↓	Zhao et al. (2017)
*Lycium barbarum* L. polysaccharide	-	Wister rats	Male	220–300 g	CCAs(15 min)/R (1 week)	intragastric administration	20 mg/kg	treatment	1 week before and after ischemia	CA1 neurons↓	Shi et al. (2017)
extract of *Ginkgo biloba* L.	-	SD rat	Male	260–280 g	MCAO (2 h)/R (24 h)	intraperitoneal injection	50 mg/kg	treatment	24 h after MCAO, 14days, q.d.	Bec-1↑,LC3-Ⅱ↑, AMPK↑, mTOR↑, ULK1↑,Parkin↑, Drp1↑,Opa1↑,Bcl-2/Bax↑	Li et al. (2019)
The combination of *Panax ginseng* C.A.Mey. and *Angelica sinensis* (Oliv.)Diels	-	SD rat	Male	250–300 g	MCAO (2 h)/R	intragastric administration	4.5.9 g/kg	treatment	3 days before MCAO,q.d., 7 days after MCAO,q.d.	Drp1↓, NLRP3↓, GSDMD↓	Hu et al. (2020)
ginsenoside Rd and LIG	BV-2 microglial cells; OGD(2 h)/R (24 h)	-	-	-	-	treated with ginsenoside Rd and LIG	Rd (0.1, 1.0, 10 μmol/l), LIG (1, 2.5, 10 μmol/l)	Prevention	2 h before OGD/R	Drp1↓, LDH↓, NLRP3↓,	Hu et al. (2020)
										GSDMD↓	

Notes: ↑, upregulate; ↓, downregulate; EC: energy charge; p-JNK1/2: Phosphorylated c-June N-terminal kinase1/2; T-NOS: total nitric oxide synthase; Fas: Frame alignment signal; FasL: frame alignment signal ligand; MMP-9: Matrix metalloproteinases 9; COX-2: Cyclooxygenase-2; CAT: catalase; GSH-Px: Glutathione peroxidase; GSSG: glutathione disulfide; Bec-1: Beclin-1; p53/Puma: 53 Up-regulatory Modulator of Apoptosis; Cleaved PARP: Cleaved poly ADP-ribose polymerase; OFR: oxygen free radical; COX: cytochrome c oxidase; eNOS: endothelial nitric oxide synthase; NLRP3: Nod-like receptor protein 3; GSDMD: Gasdermin D; ULK1, Unc-51-like kinase 1; LC3-Ⅱ, Light chain 3-Ⅱ; iNOS, inducible nitric oxide synthase; LDH, lactate dehydrogenase; GSH, glutathione; SQSTM1, Sequestosome-1; SD, Sprague-Dawley; MCAO/R, middle cerebral artery occlusion/reperfusion; q. d., once a day; bid, two times 1 day; BMECs, bone marrow endothelial cells; CCA, common carotid artery; CCA/R, common carotid artery/reperfusion; LIG, Z-ligustilide.

### 3.3 TCM compounds and their molecular mechanisms by regulating mitochondria in treating ischemic stroke

#### 3.3.1 TCM compound pretreatment in regulating mitochondria of ischemic stroke

Herbal medications have yielded many active compounds for treating ischemic stroke, and this number is increasing as research progresses. Based on published literature, we analyzed 34 TCM compounds and their molecular mechanisms in regulating mitochondria in ischemic stroke. Mitochondria appeared swollen with irregular, disrupted membranes and poorly defined cristae in an MCAO rat model. However, these mitochondrial abnormalities were prevented by piperine pretreatment (Kaushik et al., 2021). *In vitro*, OGD/R induced mitochondrial fragmentation, mitochondrial enlargement, mitochondrial number reduction, and mitochondrial swelling, which could be alleviated by pretreatment with notoginsenoside R1 (Zhu et al., 2021; Liu et al., 2022), hydroxysafflor yellow A (Huang et al., 2021), and calenduloside E (Li et al., 2022b). Ginsenoside Rd and piperine pretreatment improved mitochondrial energy metabolism after cerebral I/R injury (Ye et al., 2011; Kaushik et al., 2021) whereas notoginsenoside R1 and notoginseng leaf triterpene pretreatment improved mitochondrial energy metabolism after OGD/R injury (Xie et al., 2020; Zhu et al., 2021; Liu et al., 2022). *In vivo* (Ye et al., 2011; Mukherjee et al., 2019; Zhang et al., 2019; Huang et al., 2021; Kaushik et al., 2021) and *in vitro* (Li et al., 2017; Wu et al., 2017; Zhou et al., 2017; Huang et al., 2020; Xie et al., 2020; Li et al., 2021; Huang et al., 2021; Li et al., 2022b; Ni et al., 2022; Peng et al., 2022) studies have reported that TCM compounds (e.g., piperine, ginsenoside Rd, hydroxysafflor yellow A, β-patchoulene, curcumin, ginsenoside Rb1, artemether, notoginseng leaf triterpenes, ginkgolide k, ginsenoside monomer compound k, tanshinone IIA, artemisinin, and kaempferol) inhibited oxidative stress and mPTP and upregulated MMP levels. *In vitro* studies show that atractylenolide III, ginkgolide K, calenduloside E, and kaempferol decreased Drp1 translocation from the cytosol to the outer mitochondrial membrane, reduced its phosphorylation at Ser616, and enhanced its phosphorylation at Ser637 (Wu et al., 2017; Zhou et al., 2017; Zhou et al., 2019; Li et al., 2022b). In addition, ginsenoside Rb1 inhibits astrocyte activation and promotes the transfer of astrocytic mitochondria to neurons against ischemic stroke *in vitro* (Ni et al., 2022). Chrysophanol and ginsenoside monomer compound K decreased the level of mitochondrial autophagy in MCAO mice after I/R injury and in neurons after OGD/R injury, respectively (Huang et al., 2020; Cui et al., 2022) by inhibiting the AMPK/mTOR pathway (Huang et al., 2020). In contrast, kaempferol potentiated autophagy in primary neurons after OGD/R injury (Wu et al., 2017). Much evidence *in vivo* (Ye et al., 2011; Mukherjee et al., 2019; Zhang et al., 2019; Kaushik et al., 2021) and *in vitro* (Chen et al., 2017; Li et al., 2017; Zhou et al., 2017; Huang et al., 2020; Huang et al., 2021; Li et al., 2022b; Peng et al., 2022) suggests that TCM compounds (e.g., piperine, ginsenoside Rd, β-patchoulene, curcumin, hydroxysafflor yellow A, ginkgolide K, ginsenoside monomer compound K, tanshinone IIA, calenduloside E, artemisinin, and paeoniflorin) have protective effects against cerebral I/R injury or OGD/R injury by inhibiting mitochondria-mediated apoptosis.

**FIGURE 4:**
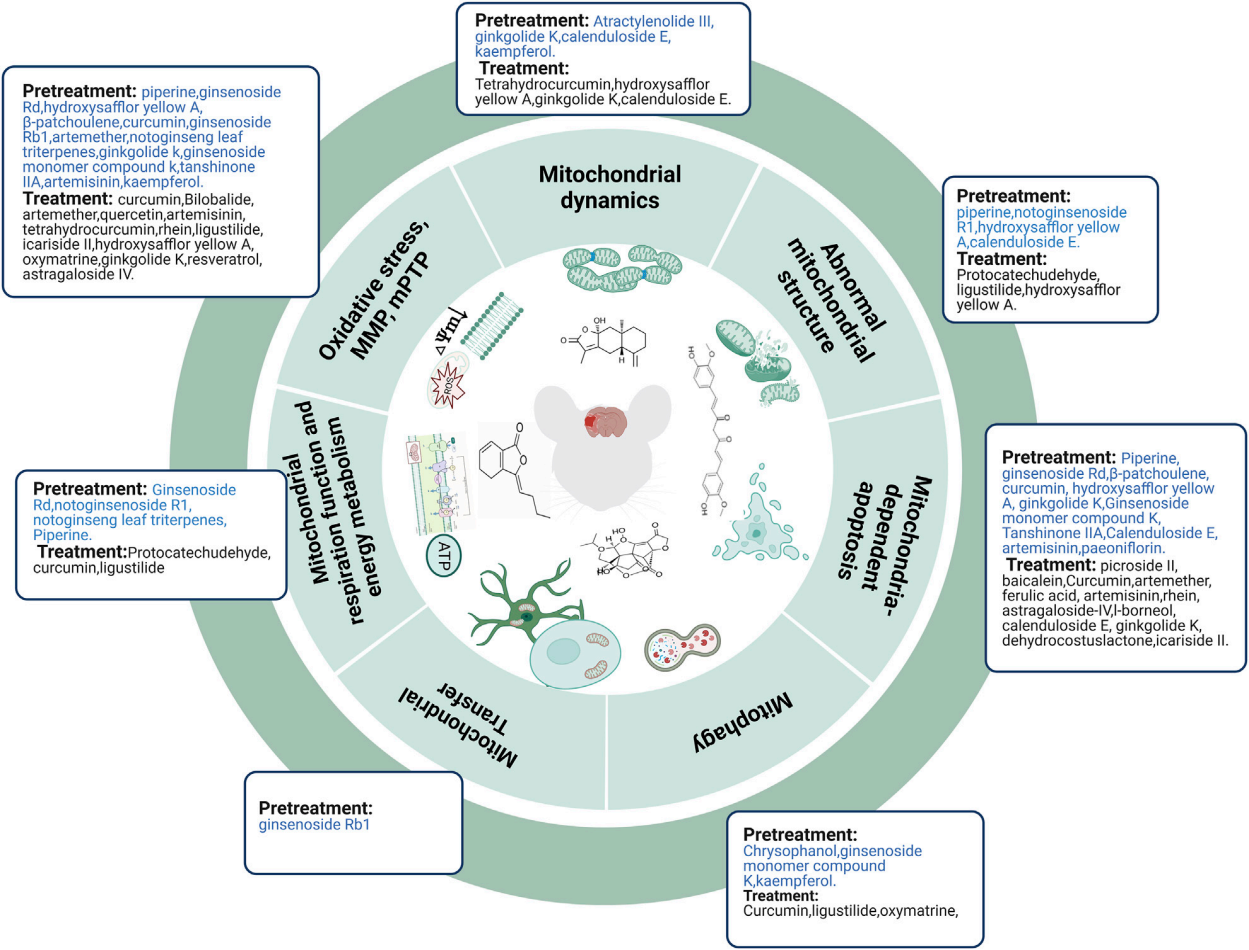
TCM compounds prevented and treated ischemic stroke by regulating mitochondria. Abbreviations: mPTP, mitochondrial permeability transition pore; MMP, mitochondrial membrane potential.

In summary, TCM compounds can alleviate abnormal mitochondrial structure, improve mitochondrial energy metabolism, decrease the expression and translocation of Drp1, reduce oxidative stress, mPTP, and mitochondria-dependent apoptosis, upregulate MMP, and promote the transfer of astrocytic mitochondria to neurons to prevent ischemic stroke. However, mitophagy results remain controversial and require further investigation (Figure 4).

#### 3.3.2 The effect of TCM compounds after ischemic stroke in regulating mitochondria

Protocatechudehyde and ligustilide improved mitochondrial morphology after cerebral I/R injury *in vivo* (Zeng et al., 2021a; Mao et al., 2022) whereas hydroxysafflor yellow A maintained mitochondrial morphology after OGD injury *in vitro* (Chen et al., 2019). Protocatechudehyde, curcumin, and ligustilide protect against cerebral ischemic injury by improving mitochondrial energy metabolism (Wang and Xu, 2020; Zeng et al., 2021a; Mao et al., 2022). Curcumin could also alleviate OGD injury by improving mitochondrial energy metabolism (Wang and Xu, 2020). Evidence from *in vivo* studies (Zhang et al., 2017; Zhao et al., 2018b; Mondal et al., 2019; Wang and Xu, 2020; Li et al., 2021; Cen et al., 2022; Mao et al., 2022; Peng et al., 2022) and *in vitro* studies (Feng et al., 2018; Liu et al., 2018; Chen et al., 2019; Xiang et al., 2019; Xue et al., 2019; Wang and Xu, 2020; Wei et al., 2021; Ye et al., 2021; Mao et al., 2022) demonstrated that, following cerebral I/R injury or OGD injury, TCM compounds (e.g., curcumin, bilobalide, artemether, quercetin, artemisinin, tetrahydrocurcumin, rhein, ligustilide, icariside II, hydroxysafflor yellow A, oxymatrine, ginkgolide K, resveratrol, and astragaloside IV) alleviated oxidative stress, inhibited mPTP, and upregulated MMP levels. Mfn-1 and Drp-1 downregulation after cerebral I/R injury was restored by tetrahydrocurcumin treatment (Mondal et al., 2019) whereas Drp-1 downregulation and Opa1 upregulation after OGD injury were restored by hydroxysafflor yellow A treatment (Chen et al., 2019). However, treating mice with ginkgolide K and calenduloside E prevents Drp1 translocation to the mitochondria and attenuates mitochondrial dysfunction after MCAO (Zhou et al., 2017; Li et al., 2022b). Curcumin and ligustilide enhanced cerebral I/R- or OGD-induced mitophagy (upregulating PINK1, Parkin, the colocalization of LC3B and mitochondrial markers, and the ratio of LC3-II to LC3-I) *in vivo* and *in vitro* (Wang and Xu, 2020; Mao et al., 2022). However, oxymatrine attenuates excessive autophagy (downregulating LC3 and Beclin-1) *in vivo* and *in vitro* by activating the PI3k/Akt pathway (Wei et al., 2021). Additionally, picroside II attenuated cerebral I/R injury by inhibiting EndoG release from the mitochondria into the cytoplasm (Li et al., 2018) Similarly, baicalein treatment decreased cerebral I/R injury by inhibiting nuclear translocation of AIF in cerebral I/R rats (Li et al., 2020). Many *in vivo* (Zhang et al., 2017; Zhao et al., 2018b; Cheng et al., 2019; Yin et al., 2020; Li et al., 2021; Zhang et al., 2021; Li et al., 2022b; Peng et al., 2022) and *in vitro* (Zhao et al., 2018a; Feng et al., 2018; Liu et al., 2018) studies have shown that TCM compounds (e.g., curcumin, artemether, ferulic acid, artemisinin, rhein, astragaloside IV, l-borneol, calenduloside E, ginkgolide K, dehydrocostus lactone, and icariside II) exert protective effects in MCAO animal models or OGD/R cell models by inhibiting BAX/BCL2, caspase-9, caspase-3, cleaved caspase-3, caspase-8, Cyt-c, Bid, Apaf-1, Bad, and p53 (Figure 4).

Through in-depth comparative analysis, TCM compounds provided during pretreatment and treatment could alleviate abnormal mitochondrial structure; inhibit oxidative stress, mPTP opening, and Drp1 translocation to the mitochondria; and improve mitochondrial energy metabolism and MMP. TCM compounds provided pretreatment could promote the transfer of astrocytic mitochondria to neurons and potentiate autophagy and also decrease the level of mitochondrial autophagy by inhibiting the AMPK/mTOR pathway. Meanwhile, TCM compounds during treatment could maintain the dynamic balance between mitochondrial fission and fusion, inhibit mitochondrial autophagy by activating the PI3k/Akt pathway, promote mitophagy by activating PINK1/Parkin, inhibit Endo G and AIF release from mitochondria into the cytoplasm, and further attenuate mitochondria-mediated apoptosis. The specific mechanisms of these compounds *in vivo* and *in vitro* are shown in Table 3; Figure 3.

**TABLE 3:** The molecular mechanism of TCM compounds in the treatment of ischemic stroke by targeting mitochondria.

Agents	Sources	Cell lines and cell models	Animals	Gender	Weight	Animal model	Routes	Dose	Prevention/treatment	Time periods	Mechanisms	References
Piperine	*Piper nigrum* L.	-	Wistar rats	Male	250–300 g	tMCAO (90 min)/R (22.5 h)	take orally	10 mg/kg	Prevention	15 days before tMCAO, qd	Cyt-c↓, caspase 3↓, Bax↓, Bcl-2↑, BDNF↑, CREB↑	Kaushik et al. (2021)
Notoginsenoside R1	*Panax notoginseng* (Burk.) F. H. Chen	HBMEC cells; OGD(2.5 h)/R (12 h)	-	-	-	-	treated with Notoginsenoside R1	6.26–100 μM	Prevention	12 h before OGD/R	NICD↓, DLL4↓, Hes1↓, Hey1↓	Zhu et al. (2021)
Notoginsenoside R1	*Panax notoginseng* (Burk.) F.H.Chen	Neuro2a cells, OGD(5% CO_2_ and 95% N_2_, 2h; 10% CCK-8 solution for 2 h)	-	-	-	-	treated with Notoginsenoside R1	5, 10, 20, 100 and 200 μM	Prevention	Before the OGD	cell viability↑, MMP↑, Atp12a↑, Atp6v1g3↑	Liu et al. (2022)
Hydroxysafflor yellow A	*Carthamus tinctorius* L.	Primary BMECs; OGD(2 h)/R (24 h)	-	-	-	-	treated with Hydroxysafflor yellow A	80 um	Prevention	2 h or 30 min before OGD/R	MMP↑, ROS↓, mPTP↓	Huang et al. (2021)
Calenduloside E	*Aralia elata* (Miq.) Seem.	The HT22 cells; OGD(0, 2, 4, 6, or 8 h)/R (24 h)	-	-	-	-	treated with Calenduloside E	1, 2, 4, 8 μg/mL	Prevention	4 h before OGD/R	Drp1↓, p-Drp1(Ser637)↑, ROS↓, Ca^2+^↓, Bax↓, Cleaved-caspase3↓, Cleaved-caspase9↓, Cyt-c↓,Bcl-2↑, caspase3↑, caspase9↑	Li et al. (2022b)
Ginsenoside Rd	*Panax ginseng* C. A. Mey.	-	SD rat	Male	270–320 g	MCAO/R (4/24 h)	Intraperitoneal Injections	50 mg/kg	Prevention	30 min before MCAO	ETC.,↑, complex I↑, complex III↑, complex IV↑, MMP↑, ROS↓	Ye et al. (2011)
Ginsenoside Rd	*Panax ginseng* C. A. Mey.	Non-synaptosomal mitochondria; OGD/R	-	-	-	-	-	-	-	-	MMP↑, ROS↓, cleaved caspase-3↓, Cyt-c↓, AIF↓	Ye et al. (2011)
Hydroxysafflor yellow A	*Carthamus tinctorius* L.	-	SD rat	Male	240–250 g	MCAO/R (24 h)	Intravenous Injections	5 mg/kg	Prevention	30 min before MCAO	ROS↓, Cyt-c↓, ATP↑, mPTP↓, Cyp D↓, MEK↓, ERK↓	Huang et al. (2021)
β-patchoulene	*Pogostemon cablin* (Blanco)Benth.	-	SD rat	Male	80–120 g	MCAO (2 h)/R (24 h)	Intravenous Injections	10 mg/kg	Prevention	Pretreatment for 1 h	Bax/Bcl-2↓, casapase-3↓, MMP↑, SOD↑, GSH-px↑	Zhang et al. (2019)
Curcumin	*Curcuma Longa* L.	-	SD rat	Male	415–440 g	CIR(30 min)/R (6 h)	Intragastric administration	5 mg/kg	Prevention	24 h before the induction of CIR	ROS↓, SDH↑, NADH↓, SOD↑, CAT↑	Mukherjee et al. (2019)
Notoginseng leaf triterpenes	*Panax notoginseng* (Burk.) F. H. Chen	SH-SY5Y cells; OGD/R	-	-	-	-	treated with Notoginseng leaf triterpenes	1.56–100 μg/mL	Prevention	24 h before OGD/R	ROS↓, MMP↑, ATP↑, NAD^+^↑, NADH↑, SIRT1/2/3↑, NAMPT↑, p-Foxo3a↑, PGC-1α↑, MnSOD↑	Xie et al. (2020)
Ginsenoside Rb1	*Panax ginseng* C. A. Mey.	Primary astrocytes; OGD(4 h)/R (1 h)	-	-	-	-	treated with Ginsenoside Rb1	0.1, 1, 10 µm	Prevention	before OGD/R	ROS↓, LDH↓, GS↑, GAPDH↓, GSH↑, NADPH↑	Ni et al. (2022)
Artemether	*Artemisia annua* L.	PC12 cells; OGD(2, 4, 6, 8 h)/R (16, 18, 20, 22 h)	-	-	-	-	treated with Artemether	10–100 μM	Prevention	2 h before OGD/R	ROS↓, MMP↑, Bax/Bcl-2↓	Li et al. (2021b)
Ginkgolide K	*Ginkgo biloba* L.	neuroblastoma Neuro2a cells; OGD(4 h)/R (1 h)	-	-	-	-	treated with Ginkgolide K	40 μM	Prevention	4 h before OGD/R	ROS↓, Drp1↓, Calcein-AM↑, mPTP↓, GSK-3β↓, MMP↑, Ca^2+^↓, Cyt-c↓, p-Drp1(Ser637)/Drp1↑, Drp1/COX-4↓	Zhou et al. (2017)
Ginsenoside monomer compound K	*Panax ginseng* C. A. Mey.	PC12 cells; OGD(2, 8 h)/R (4–24 h)	-	-	-	-	treated with Ginsenoside monomer	2, 4, 8 µM	Prevention	Pretreatment for 48 h	ROS↓, Ca^2+^↓, MMP↑, Bcl-2/Bax↑, Cleaved PARP↓, Atg5↓, LC3-II↓, Atg7↓, P-AMPK/AMPK↓, P-mTOR/mTOR↑	Huang et al. (2020)
							compound K					
Tanshinone IIA	*Salvia miltiorrhiza* Bge.	SH-SY5Y cells; 10 μL L-glutamate, 24 h	-	-	-	-	treated with Tanshinone IIA	2.5–10.0 μM	Prevention	Pretreatment for 24 h	ROS↓, MDA↓, Xanthine oxidase↓, SOD↑, CAT↑, MMP↑,ATP↑, Bcl-2↑, Bax↓, cleaved caspase-3↓, JNK↓, p38 MAPK↓	Li et al. (2017)
Artemisinin	*Artemisia annua* L.	PC12 cells; OGD(4 h)/R (20 h)	-	-	-	-	treated with Artemisinin	6.25–50 μM	Prevention	Pretreatment for 2 h	ROS↓, MMP, ERK1/2/CREB↑, Cyt-c↓, caspase 3↓, LDH↓	Peng et al. (2022)
Kaempferol	*Kaempferia galanga* L.	neuroblastoma Neuro2a cells; OGD(2 h)/R (2 h)	-	-	-	-	treated with Kaempferol	10 μM	Prevention	before OGD/R	SDH↓, Drp1↓, p-Drp1(Ser637)/Drp1↑, PAS/Drp1↑, Akt↑, PAS↑, mPTP↓, MMP↑, LC3-II/I↑, p62↑, Atg5↓	Wu et al. (2017)
Chrysophanol	*Rheum palmatum* L.	-	Kunming mice	Male	18–22 g	CCA(5 min)/R (24 h)	Intraperitoneal Injections	0.1, 1, 10 ml/kg	Prevention	Pretreatment for 10 days, 30 min before CCA	LC3B-II↓, LC3B-I↓, NIX↓, LC3B↓, LC3B-II/LC3B-I↓	Cui et al. (2022)
Atractylenolide III	*Atractylodes macrocephala* Koidz.	BV2 microglial cells; OGD/R (48)	-	-	-	-	treated with Atractylenolide III	0.01–100 μM	Prevention	cells were incubated with Atractylenolide III, followed by treatment with OGDR for 48 h	p-JAK2A↓, P-STAT3↓, P-Drp1 (Ser616)↓, P-Drp/Drp1↓, Drp/COX-4↓	Zhou et al. (2019)
Paeoniflorin	*Paeonia lactiflora* Pall.	PC12 cells; glutamate-induced (24 h)	-	-	-	-	treated with Paeoniflorin	100, 200, 300 μ M	Prevention	Pretreatment for 24 h	LDH↓, Bax↓, p-Bad↓, Bcl-2↑, Bcl-xL↑, caspase-3↓, caspase-9↓, cleaved PARP↓	Chen et al. (2017)
L-borneol	*Cinnamomum camphora* (L.) Presl	-	SD rat	Male	240–280 g	pMCAO	Intragastric administration	0.2, 0.1 and 0.05 g/kg	treatment	for 2 days before model establishment and for 1 day after model establishment	Cyt-c↓, Apaf-1↓, Bad↓, cleaved Caspase-3↓,Bcl-2↑, MEP↓, IDH2↓, MCU ↓, Apaf-1↓	Zhang et al. (2021)
Curcumin	*Curcuma Longa* L.	-	SD rat	Male	-	MCAO	Intraperitoneal Injections	100 mg/kg	treatment	once at the onset of cerebral reperfusion	ROS↓, MMP↑, ATP↑, LC3B↑, LC3-II↑	Wang and Xu (2020)
Curcumin	*Curcuma Longa* L.	Cortical Neurons; OGD(5% CO_2_ and 95% N_2_, 2 h)/R (at normal conditions, 24 h)	-	-	-	-	treated with Curcumin	5 μM	treatment	once at the stage of reoxygenation	ROS↓, MMP↑, ATP↑, LC3-II/LC3-I ↓	Wang and Xu (2020)
Protocatechudehyd	*Acacia catechu* (L. f.) Willd.	-	SD rat	Male	260 ± 20 g	tMCAO (30min)/R (7 days)	Intravenous Injections	20, 40 and 80 mg/kg	treatment	6 h after reperfusion, treatment once daily for 1 week	infarct volume↓, cell death↓, PDK1^+^↓, pPDHA1↓, acetyl CoA↑, ATP↑	Zeng et al. (2021a)
Ligustilide	*Ligusticum chuanxiong* Hort.	-	SD rat	Male	240–280 g	MCAO (2 h)/R (72 h)	Intraperitoneal Injections	10 and 20 mg/kg	treatment	at the onset of reperfusion, qd, for 3 days	mt-Atp6/Rpl13↓, Tomm20↓, COX4I1↓, p62↓, LC3-II/LC3-I↑, PINK1↑, Parkin↑, ROS↓, Na^+^ -K^+^ -ATPase↑	Mao et al. (2022)
Ligustilide	*Ligusticum chuanxiong* Hort.	HT-22 cells; OGD(5%CO_2_ and 95% N_2_, 2 h)/R (a three-gas incubator, 24 h)	-	-	-	-	treated with Ligustilide	20 μM	treatment	at the time of reperfusion	Parkin↑, PINK1↑, LC3-II/LC3-I↑, ROS↓	Mao et al. (2022)
Hydroxysafflor yellow A	*Carthamus tinctorius* L.	Similar to the primary mouse neuronal cells; OGD(5% CO_2_ and 95% N_2_, 12 h/R (5% CO_2_ at 37°C for 20 h)	-	-	-	-	treated with Hydroxysafflor yellow A	1 and 10 μM	treatment	exposed to OGD for 120 min, and treated with Hydroxysafflor yellow A for 20 h	c-cleaved Caspase-3↓, p-Akt↑, BCL2 ↑, Nerve nucleus↑, phenylalanine↓, Got1↑, ROS↓, Drp1↑	Chen et al. (2019)
Curcumin	*Curcuma Longa* L.	-	albino rats	Male	180–200 g	MCAO (30min)/R	Intraperitoneal Injections	25 mg/kg	treatment	Single dose after reperfusion	Bax↓, Bcl-2↑, p53↓, Sirt1↑, IL-6↓, TNF-α↓, MMP↓	Zhang et al. (2017a)
Quercetin	*Eucommia ulmoides* Oliv.	-	SD rat	Male	250–300 g	MCAO (2 h)	Intraperitoneal Injections	5 mg/Kg Quercetin	treatment	Two hours after MCAO	ROS↓, H_2_O_2_↓, MDA↓, GSH-Px↑, CAT↑, SOD ↑	Cen et al. (2022)
							Intravenous Injections	0.75, 2.5, 5 and 7.5 mg/Kg HA-QT				
Tetrahydrocurcumin	*Curcuma Longa* L.	-	C57BL/6J mice	Male	28–33 g	MCAO (40 min)/R (72 h)	Intraperitoneal Injections	25 mg/kg	treatment	after 4 h of ischemia. 1 time every day, lasting for 3 days	Blood-brain Barrier permeability↓, MnSOD↑, ATP↑, LC3-II↓, Mfn-1↑, Drp-1↑, DNMT1↓, DNMT3a↓, DNMT↓	Mondal et al. (2019)
Icariside II	*Epimedium brevicornu* Maxim.	PC12 cells; OGD(5% CO_2_ and 95% N_2_, 2 h)/R (5% CO_2_ and 95%air, 24 h)	-	-	-	-	treated with Icariside II	12.5, 25 and 50 μM	treatment	After deprivation of glucose and hypoxia, for 24 h	LDH↓, ROS↓, nucleus Nrf2↑, cytoplasm Nrf2 ↓, Bcl-2↑, Bax↓, Caspase-3↑, SIRT3↑, IDH2↑	Feng et al. (2018)
Oxymatrine	*Sophora flavescens* Ait.	-	SD rat	Both	12–17 g	Rice-Vannucci (hypoxia for 2.5 h)	Intraperitoneal Injections	120 mg/kg	treatment	After 48 h modeling, injected at 12 h intervals for 2 days	Beclin-1↓, LC3↓, P62↑, p-PI3K↑, p-Akt↑	Wei et al. (2021)
Oxymatrine	*Sophora flavescens* Ait.	The primary hippocampal neurons; OGD(5% CO_2_ and 95% N_2_, 2 h)/R (at normal conditions, 24 h on day 7)	-	-	-	-	treated with Oxymatrine	5 μ g/ml	treatment	After the OGD, for 24 h	neuronal apoptosis↓, Cell viability↑, Beclin-1↓, LC3↓, P62↑, PI3K ↑, Akt ↑, mTOR↑	Wei et al. (2021)
Astragaloside IV	*Astragalus membranaceus* (Fisch.) Bge.	Fetal cerebral cortical neuron; OGD(1%O_2_, 5% CO_2_, and saturated humidity, 3 h)/R (95% air and 5% CO_2_, 24 h)	-	-	-	-	treated with Astragaloside IV	25, 12.5 and 6.25 μmol/L	treatment	At the start of OGD, throughout the OGD and reoxygenation	LDH↓, Caspase-3↓, MMP↑, ATP ↓, ROS↑, PKA/CREB↑	Xue et al. (2019)
Ginkgolide K	*Ginkgo biloba* L.	SH-SY5Y cells; OGD(5% CO_2_ and 95% N_2_, 4 h)/R (5% CO_2_ and 95% O_2_,1and 24 h)	-	-	-	-	treated with Ginkgolide K	12.5, 25 and 50 μg/ml	treatment	After OGD 4 h, for 1 h	cell viability ↑, ROS↓, MMP ↓, p-p38↓, p -JNK↓, p-p53 ↓, p-c-Jun ↓, Bax↓, Bcl-2↑, cleaved Caspase-9↓, c-cleaved Caspase-3↓,	Liu et al. (2018)
Resveratrol	*Morus alba* L. or *Polygonum cuspidatum* Sieb. et Zucc.	Rat cortical neurons,OGD(1%O_2_, 5% CO_2_, and saturated humidity, 4 h)/R (at normal conditions, 2 h)	-	-	-	-	treated with Resveratrol	1–30 μM(10 μM)	treatment	After the OGD	Caspase-3↑, ROS↓, MMP↑, LC3B-II↓, TIMM23↑, TOMM20↑, PinK1↑, Parkin↑	Ye et al. (2021)
Bilobalide	*Ginkgo biloba* L.	Astrocytes; OGD(5% CO_2_ and 95% N_2_, 2.5 h)/R (at normal conditions, 3, 6, and 12 h)	-	-	-	-	treated with Bilobalide	25, 50, and 100 μ M	treatment	After the OGD	ROS ↓, MMP↑, MnSOD↑	Xiang et al. (2019)
Picroside II	*Picrorhiza scrophulariiflora* Pennell	-	Wistar rat	Male	240–260 g	MCAO (2 h)/R (24 h)	Intraperitoneal Injections	20 mg/kg	treatment	2 h after MCAO	Infarct volume↓,VDAC1↓, EndoG↓, ROS↓	Li et al. (2018b)
Baicalein	*Scutellaria baicalensis* Georgi	-	SD rat	Male	240–260 g	MCAO (60min)/R (7 days)	Intragastric administration	100 mg/kg	treatment	After 60 min MCAO,qd, for 7days	NRP1↓, Cyt-c↓, PARP-1↓, AIF↓	Li et al. (2020)
Ferulic acid	*Cinnamomum cassia* Presl	-	SD rat	Male	300–350 g	pMCAO	Intravenous Injections	60, 80 and 100 mg/kg	treatment	after MCAO	p -Akt/Akt ↑, p-mTOR/mTOR ↑, Bcl-2/Bax ↑, Cyt-c↓, c-cleaved Caspase-3↓, TUNEL-immunoreactive cells↓	Cheng et al. (2019)
Astragaloside-IV	*Astragalus membranaceus* (Fisch.) Bge.	-	SD rat	Male	280 ± 20 g	MCAO (60min)/R (7 days)	Intragastric administration	12.5, 25 and 50 mg/kg	treatment	after the reperfusion, qd. for 7days	infarct volume↓, Fas↓, FasL↓, Bcl-2/Bax ↑, Caspase-8↓, Cyt-c↓, Bid↓, Caspase-3↑, PARP-1↓	Yin et al. (2020)
Dehydrocostuslactone	*Aucklandia lappa* Decne.	hippocampal slice; OGD/R	-	-	-	-	treated with Dehydrocostuslactone	1,5, 10 µM	treatment	within the OGD/R period	LDH↓, Bcl-2↑, Bax↓, Cyt-c↓, apaf-1↓, caspase-9↓, caspase-7↓, caspase-3↓, SQSTM1↓, Lc3↓	Zhao et al. (2018a)
Rhein	*Rheum palmatum* L.	-	SD rat	Male	260–300 g	MCAO (2 h)/R (72 h)	take orally	25, 50, 100 mg/kg	treatment	after MCAO/R, qd for 3 days	MDA↓, SOD↑, GSH-px↑, CAT↑, Bax↓, Bcl-2↑, caspase-9↓, caspase-3↓, cleaved caspase-3↓	Zhao et al. (2018b)

**Notes:** ↑, upregulate; ↓, downregulate; SD, Sprague-dawley; tMCAO, transient Middle Cerebral Artery Occlusion; qd, once a day; BDNF, brain derived neurotrophic factor; CREB, cAMP, response element binding protein; NICD, Notch1 intracellular domain; CCK-8, Cell Counting Kit-8; Atp6v1g3, ATPase H + Transporting V1 Subunit H; BMECs, Brain Microvascular Endothelial Cells; p-Drp1(Ser637), Phosphorylated Drp1; ETC., electron transport chain; Cyp D, Cyclophilin D; MEK, Mitogen-activated protein kinase; ERK, extracellular regulated protein kinases; ERK1/2, Extracellular regulated protein kinases 1/2; GSH-px, glutathione-peroxidase; CIR, Cerebral ischemia-reperfusion; SDH, succinate dehydrogenase; CAT, catalase; NAD, nicotinamide adenine dinucleotide; SIRT1/2/3, sirtuin 1/2/3; NAMPT, nicotinamide phosphoribosyltransferase; p-Foxo3a, Phosphorylated Foxo3a; MnSOD, mitochondrial superoxide dismutase; LDH, lactate dehydrogenase; GS, glutamine synthetase; GAPDH, Glyceraldehyde-3-phosphate dehydrogenase; GSH, glutathione; NADPH, nicotinamide adenine dinucleotide phosphate; GSK-3β, Glycogen synthase kinase-3β; COX-4, Cyclooxygenase-4; Atg5/7, Autophagy-related gene proteins 5/7; LC3-I/Ⅱ, Light chain 3I/Ⅱ; P-AMPK, Phosphorylation of AMP-activated protein kinase; P- mTOR, phosphorylation of mammalian target of rapamycin; JNK, c-Jun N-terminal kinase; PAS, phosphorylated akt consensus sequence; LC3B-I/II, light chain 3B I/II; LC3B, light chain 3B; p-JAK2A, Phosphorylated adipocyte-Specific Deletion of Janus Kinase 2; P-STAT3, Transcription 3; IDH2, isocitrate dehydrogenase2; MCU, mitochondrial calcium uniporter; pPDHA1, phosphonated Pyruvate Dehydrogenase E1 Subunit Alpha 1; acetyl CoA, acetyl coenzyme A; mt-Atp6, Mitochondrially Encoded ATP, Synthase Membrane Subunit 6; Rpl13, Ribosomal Protein L13; COX4I1, cytochrome c oxidase subunit 4I1; Got1, glutamic oxaloacetic transaminase1; IL-6, Interleukin-6; TNF-α, Tumor necrosis factor α; HA-QT, hyaluronic acid- Quercetin; DNMT1, DNA (cytosine-5-)-methyltransferase 1; DNMT3a, DNA (cytosine-5-)-methyltransferase 3a; DNMT, DNA (cytosine-5-)-methyltransferase; Nrf2, nuclear factor erythroid 2-related factor 2; LC3, Light chain 3; p-PI3K, Phosphorylation of phosphoinositide 3 kinase; p-Akt, Phosphorylation of protein kinase B; PKA, protein kinase A; TIMM23, Translocase Of Inner Mitochondrial Membrane 23; Tomm20, translocase of outer mitochondrial membrane 20 homolog; VDAC1, voltage-dependent anion channel 1; EndoG, endonuclease G; NRP1, Neuropilin-1; PARP, poly (ADP-ribose) polymerase; TUNEL, Terminal deoxynucleotidyl transferase mediated dUTP, biotin nick end labeling; Fas, Frame alignment signal; FasL, frame alignment signal ligand; SQSTM1, Sequestosome-1; HBMEC, human brain microvascular endothelial cells; Calcein-AM, calcein acetoxymethyl ester; Atp12a, ATPase H+/K+ transporting non-gastric alpha2 subunit; Na^+^–K^+^-ATPase, Sodium potassium ATPas; CCA, common carotid artery; PMCAO, perminent Middle Cerebral Artery Occlusion; HBMEC, human brain microvascular endothelial cells.

### 3.4 TCM prescription and its molecular mechanisms by regulating mitochondria in treating ischemic stroke

#### 3.4.1 TCM prescription pretreatment for regulating mitochondria in ischemic stroke

Ischemic strokes are usually treated using TCM prescriptions owing to TCM’s overall concept of TCM and syndrome differentiation-based treatment. In MCAO rats, pretreatment with Xiao-Xu-Ming decoction improved the abnormal mitochondrial ultrastructure (Lan et al., 2018). Pretreatment with Buyang Huanwu decoction prevents H_2_O_2_-induced ultrastructural disruption of mitochondria in human umbilical vein endothelial cells, whereas Guhong injection preconditioning preserves mitochondrial morphology during OGD injury (Shen et al., 2016; Zhou et al., 2021). In human umbilical vein endothelial cells and primary cultured cortical neurons, H_2_O_2_ decreased ATP production, MDA levels, and MMP levels while increasing ROS and SOD levels, which could be reversed by pretreatment with Buyang Huanwu Decoction, Zhenbao pill, and YiQiFuMai Powder injection (Shen et al., 2016; Xu et al., 2017; Jia et al., 2021). In brain microvascular endothelial cells and primary cortical neurons, OGD/R induced MMP loss and oxidative stress injury, which could be alleviated by pretreatment with Guhong injection, Xingxiong injection, and Naoxintong capsules (Wang et al., 2021; Zhou et al., 2021; Zhu et al., 2022). Danhong injection pretreatment improved mitochondrial energy metabolism after OGD/R injury (Orgah et al., 2019). Xu et al. demonstrated that pretreatment with YiQiFuMai powder ameliorated H_2_O_2_-induced neuronal apoptosis by inhibiting mitochondrial dysfunction and PKCδ/Drp1-mediated excessive mitochondrial fission (Xu et al., 2017). Pretreatment with Xiao-Xu-Ming decoction and Zhenbao pill-containing serum exerted neuroprotective effects in MCAO rats and H_2_O_2_-induced vascular endothelial cells, respectively, by inhibiting mitophagy (Lan et al., 2018; Jia et al., 2021). In addition, Zhenbao pill-containing serum represses cell apoptosis by inhibiting autophagy (Jia et al., 2021). Xiao-Xu-Ming decoction inhibited the translocation of Smac/Diablo from the mitochondria to the nucleus, increased the level of cytoplasmic c-IAP1, and further inhibited ischemia-induced neuronal apoptosis (Lan et al., 2014). To date, many *in vivo* studies (e.g., Ershiwei Chenxiang pills, Pien-Tze-Huang and Xiao-Xu-Ming decoction) (Lan et al., 2014; Zhang et al., 2018; Hou et al., 2020) and *in vitro* studies (e.g., Buyang Huanwu decoction, Guhong injection, Xingxiong injection, YiQiFuMai powder injection, and Zhenbao pill) (Shen et al., 2016; Zhou et al., 2021; Zhu et al., 2022) have revealed that traditional Chinese prescriptions protect mitochondria from ischemic injury and inhibit the mitochondria-dependent apoptosis pathway.

**FIGURE 5:**
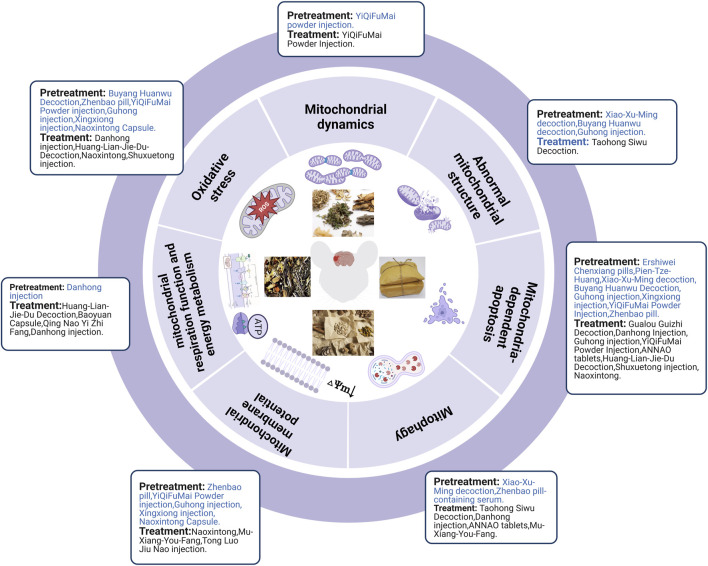
TCM prescriptions prevented and treated ischemic stroke by regulating mitochondria.

In brief, TCM pretreatment improved abnormal mitochondrial structure, inhibited oxidative stress and mitophagy, improved mitochondrial energy metabolism and MMP, and maintained the dynamic balance of mitochondrial fission and fusion. TCM prescriptions inhibited Smac/Diablo release from the mitochondria into the cytoplasm and further attenuated mitochondria-mediated apoptosis (Figure 5).

#### 3.4.2 The effect of TCM prescription after ischemic stroke in regulating mitochondria

Taohong Siwu decoction treatment for seven consecutive days decreased damage to mitochondrial structures following cerebral I/R injury (Ji et al., 2022). Treatment with Huang-Lian-Jie-Du decoction, Danhong injection, and Baoyuan capsule eliminated the inhibitory effect of cerebral I/R on mitochondrial metabolism in MCAO models (Wang et al., 2014; Zeng et al., 2021b; Du et al., 2021). *In vitro* studies have also shown that Baoyuan capsule and Qing Nao Yi Zhi Fang improved mitochondrial energy metabolism after glutamate exposure or OGD/R injury (Zhang et al., 2000; Du et al., 2021). Many *in vivo* (e.g., Danhong injection (Zeng et al., 2021b), Huang-Lian-Jie-Du-Decoction (Wang et al., 2014) and *in vitro* studies (e.g., Naoxintong (Ma et al., 2016), Shuxuetong injection (Sun et al., 2019), Mu-Xiang-You-Fang (Ma et al., 2020) and Tong Luo Jiu Nao injection (Li et al., 2014)) could downregulate mitochondrial oxidative stress levels and upregulate MMP levels in the MCAO rat and OGD/R cell models, respectively. YiQiFuMai powder injection inhibited the expression, phosphorylation, and translocation of Drp1 in oxidative stress-induced primary neurons and cerebral ischemia-injured rats, resulting in a significant improvement in cerebral infarction and neurological scores (Xu et al., 2017). Taohong Siwu decoction, Danhong injection, and ANNAO tablets upregulated the expression of autophagy markers (LC3-II/LC3-I and Beclin1) (Ji et al., 2022) and mitochondrial autophagy markers (Parkin (Orgah et al., 2019) and PINK1 (Zhang et al., 2020)) after cerebral I/R injury. Mu-Xiang-You-Fang inhibits autophagy after OGD/R-induced PC12 cell injury through the AMPK-mTOR pathway (Ma et al., 2020). Nan et al. reported that Gualou Guizhi decoction exerted its neuroprotective effects by inhibiting poly (ADP-ribose) translocation into mitochondria, thereby reducing the release and inhibiting the translocation of AIF and Endo G from mitochondria to the nucleus, which further inhibits ischemia-induced neuronal apoptosis (Nan et al., 2020). TCM prescriptions (e.g., Danhong injection (Feng et al., 2020), Guhong injection (Zhou et al., 2021), YiQiFuMai powder injection (Xu et al., 2017), ANNAO tablets (Zhang et al., 2020), and Huang-Lian-Jie-Du decoction (Wang et al., 2019)) could downregulate pro-apoptotic factors (Cyt-c, cleaved-caspase-3, cleaved-caspase-9, Bad, Bax, and Bim) and upregulate anti-apoptotic factors (Bcl-2) after cerebral I/R injury in MCAO rats. Further, TCM prescriptions (e.g., Shuxuetong injection (Sun et al., 2019) and Naoxintong (Ma et al., 2016)) also downregulated pro-apoptotic factors (e.g., Cyt-c, cleaved-caspase-3, cleaved-caspase-9, and Bax) and upregulated anti-apoptotic factors (e.g., Bcl-2) after OGD/R injury (Figure 5).

In summary, TCM prescription pretreatment and treatment could improve the abnormal mitochondrial structure, mitochondrial energy metabolism, and MMP, and inhibit oxidative stress, mitochondrial fission, and mitophagy. Moreover, TCM prescription pretreatment inhibited Smac/Diablo release from mitochondria into the cytoplasm and further attenuated mitochondria-mediated apoptosis. In contrast, TCM prescription treatment promoted mitophagy by activating PINK1/Parkin and inhibiting mitochondria-mediated apoptosis by attenuating AIF and Endo G release from mitochondria into the cytoplasm. The specific mechanisms of the TCM prescriptions *in vivo* and *in vitro* are shown in Table 4; Figure 3.

**TABLE 4:** The molecular mechanism of TCM prescription in the treatment of ischemic stroke by targeting mitochondria.

Agents	Ingredients	Cell lines and cell models	Animals	Gender	Weight	Animal model	Routes	Dose	Prevention/treatment	Time periods	Mechanisms	References
Xiao-Xu-Ming decoction	*Ephedra sinica* Stapf, *Cassia obtusifolia* L., *Paeonia lactiflora* Pall., *Ligusticum chuanxiong* Hort., *Panax ginseng* C. A. Mey., *Cnidium monnieri* (L.)Cuss., *Scutellaria baicalensis* Georgi, *Dioscorea opposita* Thunb., *Lindera aggregate* (Sims)Kos-term., *Aconitum kusnezoffii* Reichb., *Glycyrrhiza uralensis* Fisch., *Saposhnikovia divaricata* (Turcz.)Schischk., *Coptis chinensis* Franchet.	-	SD rat	Male	250–280 g	MCAO (90 min)/R (24 h)	take orally	60 g/kg	Prevention	Pretreatment 3 days, until the conclusion of the experiment. bid	Cell Injury↓, MDA↓, ATP↑,LC3B↓,VDAC1↓,LAMP1↓,Beclin1↓,p62↓	Lan et al. (2018)
Buyang Huanwu Decoction	*Astragalus membranaceus* (Fisch.)Bge., *Angelica sinensis* (Oliv.)Diels, *Paeonia lactiflora* Pall., *Ligusticum chuanxiong* Hort., *Fritillaria cirrhosa* D.Don, *Prunus persica* (L.)Batsch, *Carthamus tinctorius* L., *Atractylodes macrocephala* Koidz.	HUVECs, exposed to H_2_O_2_ (320 μ mol/l) for 6 h	-	-	-	-	Treatment with different concentrations of Buyang Huanwu Decoction	5,15 and 30 mg/ml	Prevention	pretreated for 6 h	cell viability↑, apoptosis ↓, cleaved Caspase-3↓, ROS↓, MDA↓, SOD↑,MMP↑	Shen et al. (2016)
Guhong injection	Aceglutamide, *Carthamus tinctorius* L.	Rat Brain Microvascular Endothelial Cells,OGD(1%O2,5% CO_2_, and 94%N_2_,6 h)/R (at normal conditions,6 h)	-	-	-	-	treated with different concentrations of Guhong injection	25,50 and 100 μ l/ml	Prevention	before culture under OGD for 6 h	apoptosis rate↓, MMP↓,Cyt-c↓, LDH↓,MMP-9↓, SOD↑, MDA ↓,p-Akt↑,Bax/Bcl-2↑, cleaved Caspase-3↓, Caspase-3↓	Zhou et al. (2021a)
Guhong injection	Aceglutamide, *Carthamus tinctorius* L.	-	SD rat	Male	280 ± 10 g	tMCAO (1 h)/R (7 days)	intraperitoneal injection	2.5, 5 and 10 ml/kg	treatment	after MCAO, bid for 7 days.	infarct Volume ↓, cleaved Caspase-3↓, Bcl-2/Bax↑, Cyt-c ↓	Zhou et al. (2021a)
YiQiFuMai powder injection	*Panax ginseng* C. A. Mey., *Ophiopogon japonicus* (L.f)Ker-Gawl., *Schisandra chinensis* (Turcz.)Baill.	Primary Cortical Neurons, exposed to H_2_O_2_(100 μ M)for 12 h	-	-	-	-	Treated with YiQiFuMai powder injection	25–800 μ g/ml	Prevention	pretreated for 6 h	Caspase-3↑, cleaved Caspase-3↑,ROS↓, ATP↑,MMP↑,Bcl-2 ↓, Bcl-xl↓, Bax↓,Drp1↓	Xu et al. (2017)
YiQiFuMai powder injection	*Panax ginseng* C. A. Mey., *Ophiopogon japonicus* (L.f)Ker-Gawl., *Schisandra chinensis* (Turcz.)Baill.	-	SD rat	Male	280–300 g	tMCAO (90min)/R (24 h)	intraperitoneal injection	0.957 g/kg	treatment	after 90 min of ischemia	Bcl-2↑, Bax↓, cleaved Caspase-9↓, Drp1↓	Xu et al. (2017)
Xingxiong injection	*Ginkgo biloba* L. extract, tetramethylpyrazine sodium chloride	Primary cortical neurons,OGD(5% CO_2_ and 95% N_2_, 2 h)/R (at normal conditions,24 h)	-	-	-	-	treated with Xingxiong injection	1, 2 and 4 μL/mL	Prevention	before the OGD	Caspase-3 ↓NOX↓, 4-HNE↓,8-OHdG↓	Zhu et al. (2022)
Naoxintong Capsule Combined with Guhong Injection	Aceglutamide, *Carthamus tinctorius* L., *Astragalus membranaceus* (Fisch.)Bge., *Salvia miltiorrhiza* Bge., *Paeonia lactiflora* Pall., et al.	rBMEC,OGD(1%O_2_,5% CO_2_, and 94%N_2_,4 h)/R (at normal conditions,6 h)	-	-	-	-	treated with Drug-contained rat sera	12.5,25 and 50 ml/kg, for 14 days	Prevention	At the start of OGD	MDA↓,SOD↑, The apoptotic and necrotic cells↓, MMP↑	Wang et al. (2021a)
The Zhenbao pill	*Pteria martensii* (Dunker), *Cassia obtusifolia* L., *Bos taurus* domesticus Gmelin, *Cervus elaphus* Linnaeus, *Saiga tatarica* Linnaeus, *Glycyrrhiza uralensis* Fisch., et al.	HUVECs, exposed to H_2_O_2_(500 μM) for 2 h	-	-	-	-	treated with 10% Drug-contained rat sera	0.25,0.5 and 1 g/kg, for 7 days	Prevention	pretreated for 12 h	cell viability↑, LDH↓, apoptosis↓,ROS↓,MMP↑,AKT↑,mTOR↑, cell autophagy↓, cell apoptosis↓,Bcl2↑,Beclin1↓,BAX↓, cleaved LC3 II ↓	Jia et al. (2021)
Ershi-wei Chenxiang pills	*Aquilaria sinensis* (Lour.) Gilg, *Ewgewia caryophyllata* Thunb., *Chaenomeles speciose* (Sweet) Nakai, *Myristica fragrans* Houtt., *Carthamus tinctorius* L., *Choerospondias axillaris* (Roxb.) Burtt et Hill, *Inula recemosa* Hook. f., *travertine*, *Cervus elaphus* Linnaeus, *Boswellia carterii* Birdw., *Hyriopsis cumingii* (Lea), *Aucklandia lappa* Decne., *Strychnos nux-vomica* L., *Terminalia chebula* Retz., *Lagotis brachystachya* Maxim., *Gossampinus malabarica* (DC.) Merr., *Phyllanthus emblica* L., *Dalbergia odorifera* T. Chen, *Lepus oiostolus* Hodgson, and *Bos Taurus* domesticus Gmelin.	-	SD rat	Male	260–300 g	MCAO (2 h)/R (24 h)	take orally	1.33 and 2.00 g/kg	Prevention	pretreated for 14 days,qd	cell viability↑, neuronal apoptosis ↓, Bcl-2↑, Bax↓, Caspase-3↓, Cyt-c↓, CaMK Ⅱ↓, ATF4 ↓,c-Jun↓	Hou et al. (2020)
Pien-Tze-Huang	taurine, malic acid, citric acid, notoginsenoside R1, ginsenosides Rg1, Rb1, Re, Rf, Rd, Rg2, Rg3, Rh1, muscone, cholic acid, hyodeoxycholic acid, taurocholic acid, ursodeoxycholic acid, chenodeoxycholic acid, taurochenodeoxycholic acid, tauroursodeoxycholic acid, glycodeoxycholic acid, and glycocholic acid.	-	SD rat	Male	240 ± 20 g	MCAO (1.5 h)/R (24 h)	take orally	180 mg/kg	Prevention	pretreated for 4 days,qd	IL-1β↓,IL-6↓,TNF-α↓, neuronal apoptosis↓, p-AKT↑, p-GSK-3β↑,mitochondrial Cyt-c↑, Cytosolic Cyt-c↓, cleaved Caspase-3↓, cleaved Caspase-9↓, Bax↓, Bcl-xl↑, P53↓	Zhang et al. (2018b)
Xiao-Xu-Ming decoction	*Ephedra sinica* Stapf, *Cassia obtusifolia* L., *Paeonia lactiflora* Pall., *Ligusticum chuanxiong* Hort., *Panax ginseng* C. A. Mey., *Cnidium monnieri* (L.)Cuss., *Scutellaria baicalensis* Georgi, *Dioscorea opposita* Thunb., *Lindera aggregate* (Sims)Kos-term., *Aconitum kusnezoffii* Reichb., *Glycyrrhiza uralensis* Fisch., *Saposhnikovia divaricata* (Turcz.)Schischk., *Coptis chinensis* Franchet.	-	SD	Male	250–280 g	MCAO (90 min)/R (24 h)	take orally	60 g/kg	Prevention	pretreated for 3 days,tid	apoptosis↓,p53↓,Bcl-2↑,Bax↓, Cyt-c↓, Smac/Diablo↓, cytoplasmic c-IAP1↑, Caspase-9↓, Caspase-3↓, Nissl vesicles↑, TUNEL-positive cells↓, Beclin1↑, LC3-I ↑,PINK1↑,Parkin↑	Lan et al. (2014)
Taohong Siwu Decoction	*Prunus persica* (L.) Batsch, *Carthamus tinctorius* L., *Rehmannia glutinosa* Libosch., *Paeonia lactiflora* Pall., *Angelica sinensis* (Oliv.) Diels, *Ligusticum chuanxiong* Hort.	-	SD rat	Male	220–270 g	MCAO (2 h)/R (7 days)	Intraperitoneal injection	9 g/kg	treatment	after MCAO, qd for 7 days	ROS↓, NLRP3↓, cleaved caspase 1↓, IL-1β↓,IL18↓	Ji et al. (2022)
Bao Yuan Capsule	*Cordyceps sinensis* (BerK.)Sacc., *Astragalus membranaceus* (Fisch.)Bge.var.*mongholicus* (Bge.)Hsiao, *Panax ginseng* C. A. Mey., *Panax notoginseng* (Burk.) F. H. Chen	-	C57BL/6 N mice	Male	20–25 g	MCAO (1.5 h)/R (24days)	Intragastrical administration	1,2,4 g/kg	treatment	Start on day 3 after MCAO, for 21 days	BrdU^+^/NeuN+↑,BrdU^+^/DCX↑, p-Akt↑,p-GSK-3β↑, AMPK↑,β-catenin↓, ACO2↑,SDHA↑	Du et al. (2021)
Bao Yuan Capsule	*Cordyceps sinensis* (BerK.)Sacc., *Astragalus membranaceus* (Fisch.)Bge.var.*mongholicus* (Bge.)Hsiao, *Panax ginseng* C. A. Mey., *Panax notoginseng* (Burk.) F. H. Chen	C17.2 cells,OGD(5% CO_2_ and 95% N_2_,2 h)/R (at normal conditions,2 or 48 h)	-	-	-	-	treated with Bao Yuan Capsule	200 μg/ml	treatment	treat for 48 h	ATP↑,AMP↑,ADP↓,ATP/ADP↑, DCX positive↑	Du et al. (2021)
Danhong injection	*Salvia miltiorrhiza* Bge., *Carthamus tinctorius* L.	-	SD rat	Male	260 ± 20 g	MCAO (60 min)/R (7 days)	intravenous injection	0.5,1.0 and 2.0 mL/kg	treatment	after MCAO, qd for 7days	Inhibits apoptosis↓, SOD↑,T-AOC↑, γH2AX↓,PARP1↓, AIF in nuclear↓, HSP70↑, NAD ^+^↑, pyruvate↑,HIF1α↓, PDK1↓, pPDHA1 ↓, CoA ↑, ATP ↑, ATP-dependent Na^+^ -K^+^-ATPase↑	Zeng et al. (2021b)
Huang-Lian-Jie-Du-Decoction	*Coptis chinensis* Franch., *Scutellaria baicalensis* Georgi, *Phellodendron chinense* Schneid., *Gardenia jasminoides* Ellis	-	SD rat	Male	280 ± 20 g	MCAO (2 h)/R (24 h)	intragastric administration	5 g/kg	treatment	after MCAO, qd for 10 days	infarct area↓, the metabolic disturbance↓	Wang et al. (2014a)
Danhong injection	*Salvia miltiorrhiza* Bge., *Carthamus tinctorius* L.	Primary cortical neurons, OGD(5% CO_2_ and 95% N_2_, 2 h)/R (at normal conditions,4 h)	-	-	-	-	Treated with Danhong injection	0.75,1.5,3.0 mL/kg	Prevention	incubated the cells for 20 min with Danhong injection	mitochondrial reductase activity ↑	Orgah et al. (2019)
Danhong injection	*Salvia miltiorrhiza* Bge., *Carthamus tinctorius* L.	-	SD rat	Male	250–300 g	MCAO/R	intravenous injection	0.75,1.5 and 3.0 mL/kg	treatment	After MCAO, bid for 14 days	parkin ↑	Orgah et al. (2019)
Qing Nao Yi Zhi Fang	taurine, malic acid, citric acid, notoginsenoside R1, ginsenosides Rg1, Rb1,Re,Rf,Rd,Rg2,Rg3, Rh1, muscone, cholic acid, hyodeoxycholic acid, taurocholic acid, ursodeoxycholic acid, chenodeoxycholic acid, taurochenodeoxycholic acid, tauroursodeoxycholic acid, glycodeoxycholic acid, and glycocholic acid	neuronal cells, exposed to glutamate,72 h	-	-	-	-	treated with Drug-contained rat sera	50 μl/ml drug-serum	treatment	After 72 h	ChE ↑, SOD ↑, NO↓, LDH↓, SDH ↑, MMP ↑,ATP↑,apoptosis↓	Zhang et al. (2000)
Naoxintong capsule	*Astragalusmembranaceus* (Fisch.)Bge.var.*mongholicus* (Bge.)Hsiao, *Paeonia lactiflora* Pall., *Salvia miltiorrhiza* Bge, *Angelica sinensis* (Oliv.) Diels, *Ligusticum chuanxiong* Hort., *Prunus persica* (L.) Batsch, *Carthamus tinctorius* L., *Boswellia carterii* Birdw., *Commiphora myrrha* Engl., *Spatholobus suberectus* Dunn, *Achyranthes bidentata* Bl., *Cinnamomum cassia* Presl, *Morus alba* L., *Pheretima aspergillum* (E.Perrier), *Buthus martensii* Karsch, *Hirudo nipponica* Whitman	Primary Cortical Neurons, OGD(5% CO_2_ and 95% N_2_, 4 h)/R (at normal conditions,2 h)	-	-	-	-	treated with Cerebrospinal fluid containing Naoxintong capsule (BNC)	(2.5%, 5%, and 10%) BNC, Containing Naoxintong capsule	treatment	At the start of OGD, for 4 h	cell viability↑, apoptosis ↓, Ca^2+^↓, ROS↓,NO↓,nNOS↓, mPTP Opening↓, Cyt-c↓, MMP↓, Bcl-2↑, Bax↓, Caspase-3↓, Caspase-9 ↓, p-Akt↑	Ma et al. (2016)
Shuxuetong injection	*Hirudo nipponica* Whitman, *Pheretima aspergillum* (E.Perrier)	bEnd.3, OGD(5% CO_2_ and 95% N_2_, 6 h)/R (95% air and 5% CO_2_, 18 h)	-	-	-	-	treated with Shuxuetong injection	The effective concentration of Shuxuetong injection was separately diluted 32-, 64-, and 128-times	treatment	added to cells during OGD/R	cell viability↑, dehydrogenase leakage↓, cleaved Caspase-3↓,Bcl-2↑,mitochondrial superoxide production↓, oxygen species↓,TNF-α↓,IL-6↓, NF-κB p65 ↓, p-IκBα ↓,IL-1β↓,p-IKK↓, inducible nitric oxide synthase↓, claudin-5↑	Sun et al. (2019)
Mu-Xiang-You-Fang	Aucklandia lappa Decne, Piper nigrum L, Euphorbia pekinensis Rupr, Callorhinusursins Linnaeus, Asarum heterotropoides Fr.Schmidt Var. mandshuricum (Maxim.) Kitag	PC12 cells, OGD(5% CO2 and 95% N2, 2 h)/R (5% CO_2_ and 95%O_2_,24 h)	-	-	-	-	treated with Mu-Xiang-You-Fang	1, 2, 4 μg/mL	treatment	after MCAO	LDH ↓,MMP↑, Ca^2+^↓, survival rate↑,autophagy↓, LC3 ↓,p62↑, beclin1 ↓, p-AMPK↓, ULK1↓, p-mTOR↑, p-p70s6k↑	Ma et al. (2020)
Tong Luo Jiu Nao injection	*Panax notoginseng* (Burk.) F.H.Chen, *Gardenia jasminoides* Ellis	BMECs,OGD(7% CO_2_ and 93% N_2_, 6 h)/R (at normal conditions,10 h)	-	-	-	-	treated with Tong Luo Jiu Nao injection	2 μl/ml	treatment	after MCAO	LDH↓, Ca^2+^↓, NMDAR1↓,MMP↑,Cyt-c↓, VEGF↑, PAF↓	Li et al. (2014)
ANNAO tablets	Not mentioned	-	SD rat	Male	250–270 g	MCAO (2 h)/R (1 or 7 days)	Intragastrical administration	300,600 and 1,200 mg/kg	treatment	1 h after the start of reperfusion, qd for 1 day or 7 days	infarct volumes ↓, PINK1↑, Parkin↑, Drp1↑, Cyt-c↓,Bcl-2/Bax↑, NeuN-positive neuron↑	Zhang et al. (2020b)
Gualou Guizhi Decoction	*Trichosanthes kirilowii* Maxim., *Cinnamomum cassia* Presl., *Paeoniae lactiflora* Pall*., Zingiber officinale* Rosc., *Ziziphus jujuba* Mill., *Glycyrrhiza uralensis* Fisch.	-	SD rat	Male	210–230 g	MCAO (2 h)/R (7days)	Intragastrical administration	3.6 g/kg,7.2 g/kg and14.4 g/kg	treatment	after MCAO, qd for 7 days	Nissl-Positive Cells↑,PARP-1↓, AIF↓, Endo G↓, Hsp70↑, nucleus PARP-1↓, nucleus AIF ↓, nucleus Endo G↓, mitochondria AIF↑, mitochondria Endo G↑	Nan et al. (2020)
Danhong Injection	*Salvia miltiorrhiza* Bge., *Carthamus tinctorius* L.	-	SD rat	Male	260–290 g	MCAO/R	Intravenous Injections	0.84 mL/kg	treatment	after MCAO, qd for 3 days.	Apoptosis↓, Cyt-c↓,MDM2↓,p-Akt↑, Bim↓,p53↓	Feng et al. (2020)
Huang-Lian-Jie-Du Decoction	*Coptis chinensis* Franch., *Scutellaria baicalensis* Georgi, *Phellodendron chinense* Schneid., *Gardenia jasminoides* Ellis	-	SD rat	Male	200–220 g	MCAO (1.5 h)/R (24 h)	Intraperitoneal Injections	Baicalin (5 mg/ml),jasminoidin (25 mg/ml)	treatment	After reperfusion	Bak↓	Wang et al. (2019b)

Notes: ↑, upregulate; ↓, downregulate; SD, Sprague-Dawley; MCAO/R, middle cerebral artery occlusion/reperfusion; bid, two times a day; LC3B, light chain 3B; VDAC1,voltage-dependent anion channel 1; Lamp1, Lysosome-associated membrane protein 1; HUVEC, human umbilical vein endothelial cells; H2O2, hydrogen peroxide; Caspase, cysteinyl aspartate specific proteinase; MMP-9, matrix metalloproteinase-9; LDH, lactate dehydrogenase; tMCAO, transient middle cerebral artery occlusion; NOX, NADPH, oxidases; 4-HNE, 4-Hydroxynonena; 8-OHdG,8-Hydroxydeoxyguanosine; rBMEC, brain microvessel endothelial cells; LC3-Ⅱ, Light chain 3Ⅱ; qd, once a day; CaMK Ⅱ, calmodulin-dependent protein kinase II; ATF4, Activating Transcription Factor 4; IL-1β, Interleukin-1, beta; IL-6, Interleukin-6; TNF-α, tumor necrosis factor-α; p-GSK-3β, phosphonated glycogen synthase kinase-3β; p53, protein 53; tid, three time a day; TUNEL, terminal deoxynucleotidyl transferase-mediated dUTP-biotin nick end labeling; LC3-I, Light chain 3 I; PINK1, PTEN, induced putative kinase 1; NLRP3, Nod-like receptor protein 3; IL18, Interleukin-18; BrdU+, 5-Bromodeoxyuridinc; DCX, doublecortin; ACO2, aminocyclopropanecarboxylate oxidase; SDHA, Succinate Dehydrogenase Complex Flavoprotein Subunit A; AMP, adenosine monophosphate; ADP, adenosine vdiphosphate; DCX, doublecortin; C17.2 cells, C17.2 mouse neural stem cells; T-AOC, total antioxidant capacity; PARP1, poly ADP-ribose polymerase 1; HSP70, Heat shock 70 kDa protein; NAD, nicotinamide adenine dinucleotide; CoA, coenzyme A; ChE, choline esterase; NO, nitric oxide; SDH, succinate dehydrogenase; nNOS, neuronal nitric oxide synthase; NF-κB, Nuclear factor kappa-B; p-IKK, phosphonated Inhibitor of kappa B kinase; LC3, light chain 3; p-AMPK, Phosphorylation of AMP-activated protein kinase; P-mTOR, phosphorylation of mammalian target of rapamycin; p-p70s6k, Phosphorylation of p70 ribosomal protein S6 kinase; NMDAR1, N-methyl-D-aspartic acid receptor1; VEGF, vascular endothelial growth factor; PAF, Platelet-activating factor; Drp1, Dynamin-related protein 1; MDM2, murine double minute2; p-IκBα, phosphonated inhibitor of nuclear factor kappaB; ULK1, Unc-51-like kinase 1; γH2AX, gamma H2A histone family member X; pPDHA1, phosphonated Pyruvate Dehydrogenase E1 Subunit Alpha 1.

### 3.5 The specific molecular mechanism among acupuncture, herbal extracts, TCM compounds, and TCM prescriptions in treating ischemic stroke

Acupuncture, herbal medicine, TCM compounds, and TCM prescriptions prevent and treat ischemic stroke by improving abnormal mitochondrial structure, increasing MMP levels, mitochondrial respiration function, and mitochondrial energy metabolism, decreasing oxidative stress, maintaining mitochondrial fission and fusion dynamics, promoting mitochondrial biogenesis, regulating mitophagy, and inhibiting mitochondrial-dependent apoptosis. However, the specific molecular mechanism differs among acupuncture, herbal extracts, TCM compounds, and TCM prescriptions in treating ischemic stroke. Acupuncture, herbal extract, TCM compounds, and TCM prescriptions could alleviate mitochondrial respiration function and energy metabolism by improving the electron transport chain. Acupuncture, TCM compounds, and TCM prescriptions can improve tricarboxylic acid cycle dysfunction. Additionally, TCM compounds and TCM prescriptions can improve mitochondrial respiration and energy metabolism by inhibiting the hypoxia-inducible factor 1-alpha/pyruvate dehydrogenase kinase 1(HIF1α/PDK1) pathway. Acupuncture inhibited mitochondrial fission, whereas herbal extracts and TCM prescriptions promoted mitochondrial fusion. However, the results regarding mitochondrial fission have been inconsistent in studies of herbal extracts, TCM compounds, and TCM prescriptions. We noted that TCM compounds decreased Drp1 translocation from the cytosol to the outer mitochondrial membrane by inhibiting Jak2/Stat3. In contrast, TCM prescriptions decreased Drp1 translocation from the cytosol to the outer mitochondrial membrane by inhibiting PKCδ. Acupuncture, TCM compounds, and TCM prescriptions promoted mitophagy by activating the PINK1/Parkin pathway. On the other hand, herbal extracts, TCM compounds, and TCM prescriptions suppressed mitophagy by inhibiting the AMPK/mTOR pathway.

Acupuncture could attenuate mitophagy by inhibiting the p-ULK1/FUNDC1 pathway, whereas TCM compounds could inhibit mitophagy by activating the PI3k/Akt pathway. Acupuncture, herbal extracts, TCM compounds, and TCM prescriptions can upregulate the expression of anti-apoptotic proteins in the BCL-2 family and downregulate the expression of pro-apoptotic proteins in the BCL-2 family. In addition, acupuncture prevented Smac/DIABLO and cofilin translocation from the mitochondria into the cytoplasm, whereas TCM compounds inhibited Endo G and AIF release from the mitochondria into the cytoplasm. Moreover, TCM prescriptions could inhibit Smac/DIABLO, Endo G, and AIF release from the mitochondria into the cytoplasm. Generally, the above evidence demonstrated that the specific molecular mechanism differed among acupuncture, herbal extract, TCM compounds, and TCM prescriptions in treating ischemic stroke (Figures 1, 3).

Interestingly, only TCM compounds have been reported to promote the transfer of astrocytic mitochondria to neurons in response to ischemic stroke. Emerging evidence suggests that mitochondria could serve as “help-me” signaling in response to various external stimuli and recruit neighboring cells to rescue injured cells. Removing damaged mitochondria and replacing them with healthy ones is a potential treatment for hypoxia and ischemia-related disorders, especially in the central nervous system, where mitochondria are abundant in the distal axonal synapses and dendritic protrusions. More studies can be conducted exploring the underlying therapeutic mechanism of TCM in treating ischemic stroke from the perspective of mitochondrial transfer.

## 4 Conclusion and prospects

In this review, we summarize the molecular mechanisms underlying the involvement of mitochondria in ischemic stroke. Mitochondrial function and structure play important roles in ischemic stroke, serving as crucial targets for TCM in alleviating ischemic stroke, and we have identified some key proteins and signaling pathways, as mentioned above. In addition, some issues require further clarification and improvement in future research. First, the precise molecular mechanisms underlying the effects of TCM on mitochondria in cellular and rat models of ischemic stroke remain incompletely understood. Further research is needed to elucidate the underlying mechanisms of TCM’s effects on mitochondrial structure and function in ischemic stroke, using molecular, cellular, and biochemical approaches. Second, the lack of standardized experimental designs and methods may affect the reproducibility and comparability of the results. Standardization of experimental designs and methods, including the quality control of TCM preparations, should be established to ensure the scientific rigor and reliability of future studies. Third, in the field of EA therapy for ischemic stroke, research from the perspective of mitochondria is scarce in comparison to studies on herbal extracts, compounds, and prescriptions. More rigorous and well-designed studies are urgently needed. In addition, it is critical to establish standardized EA stimulation parameters (e.g., frequency, duration, and intensity) to investigate the dose-response relationship and corresponding mechanisms from the perspective of mitochondria in future studies. Fourth, the results of mitophagy, mitochondrial fission, and mitochondrial fusion have been inconsistent among studies. These controversial results might be associated with different experimental models, different stages of ischemic stroke, and intervention modes. Further investigations are required to elucidate this. Fifth, most of the studies summarized above are based on OGD/R or MCAO ischemia models, and most MCAO ischemia models are conducted on young rats or mice. Few studies have used aged animals or models that closely mimic clinical patients who often have hypertension, hyperglycemia, or other disorders. Therefore, it is necessary to investigate the neuroprotective benefits of TCM against ischemic stroke using pseudo-clinical models (e.g., complicated models of multiple coexisting disorders), which will provide a reliable foundation for TCM’s clinical application of TCM. Sixth, most *in vitro* studies mentioned above were limited to a particular type of nerve cells. Neurovascular dysfunction induced by ischemic stroke demonstrates a combined action of multiple nerves in the brain, and investigating only one type of nerve cell is insufficient. Thus, ischemic stroke can be better understood using a cell co-culture model of neurons, microglia, and astrocytes *in vitro*. Finally, since Chinese herbs and prescriptions contain various chemical components, the precise underlying mechanisms remain unknown. Further research is required into the molecular targets and active components that contribute to the bioactivity of Chinese herbs and prescriptions in preventing and treating ischemic stroke. Furthermore, we should examine the synergic effects of the constituents and their metabolites, as well as the targeted signaling pathways in post-ischemic brains.

In conclusion, this review summarizes the recent experimental evidence of TCM in preventing and treating ischemic stroke by modulating mitochondria and identifies areas that future research should focus on. In addition, TCM has few side effects and is highly effective and specific; therefore, with adequate research, it will be widely available for ischemic stroke treatment.

## Glossary

ATP: adenosine triphosphate
TCM: traditional Chinese medicine
OGD: oxygen-glucose deprivation
ROS: reactive oxygen species
tPA: tissue plasminogen activator
mPTP: mitochondrial permeability transition pore
MMP: mitochondrial membrane potential
NAD+: nicotinamide adenine dinucleotide
MAMs: mitochondria-associated endoplasmic reticulum membranes
I/R: ischemia/reperfusion
OGD/R: oxygen-glucose deprivation/reoxygenation
mtDNA: mitochondrial DNA
PGC-1α: peroxisome proliferator-activated receptor γ coactivator-1α
AMPK: AMP-activated protein kinase
SIRT1: phosphorylation and sirtuin 1
NRF1/2: nuclear respiratory factors 1/2
TFAM: mitochondrial transcription factor A
Drp1: dynamin-related protein 1
Mfn1/2: mitofusins-1/mitofusins-2
Opa1: optic atrophy 1
BNIP3: Bcl-2/E1B-19 KD-interacting protein 3
FUNDC1: FUN14 domain containing 1
MCAO: middle cerebral artery occlusion
Endo G: endonuclease G
Apaf-1: apoptosis activating factor-1
IAPs: inhibitor-of-apoptosis proteins
AIF: apoptosis-related proteins
EA: electroacupuncture
MDA: malondialdehyde
SOD: superoxide dismutase
mTOR: mammalian target of rapamycin
Smac/Diablo: the second mitochondrion-derived activator of caspase/direct inhibitor of apoptosis-binding protein with low pI
Cyto-Cyt-c: Cyt-c in the cytoplasm
Mito-Cyt-c: mitochondrial Cyt-c
cIAP-1: -2, cellular inhibitor of apoptosis- 1,-2
XIAP: X-linked inhibitor of apoptosis protein
p-Bad: phospho-Bad
BNIP3L: NIP3-like protein X
Cyt-c: cytochrome C
HIF1α/PDK1: hypoxia-inducible factor 1-alpha/pyruvate dehydrogenase kinase 1
ROS: reactive oxygen species
